# Dynamic cerebral blood flow assessment based on electromagnetic coupling sensing and image feature analysis

**DOI:** 10.3389/fbioe.2024.1276795

**Published:** 2024-02-21

**Authors:** Zhiwei Gong, Lingxi Zeng, Bin Jiang, Rui Zhu, Junjie Wang, Mingyan Li, Ansheng Shao, Zexiang Lv, Maoting Zhang, Lei Guo, Gen Li, Jian Sun, Yujie Chen

**Affiliations:** ^1^ School of Pharmacy and Bioengineering, Chongqing University of Technology, Chongqing, China; ^2^ College of Artificial Intelligence, Chongqing University of Technology, Chongqing, China; ^3^ College of Biomedical Engineering, Army Medical University, Chongqing, China; ^4^ School of Information and Communication Engineering, Dalian University of Technology, Dalian, Liaoning, China; ^5^ Department of Neurosurgery, Southwest Hospital, Army Medical University, Chongqing, China

**Keywords:** cerebral blood flow, dynamic assessment, image features, vascular stiffness, cerebral oxygen

## Abstract

Dynamic assessment of cerebral blood flow (CBF) is crucial for guiding personalized management and treatment strategies, and improving the prognosis of stroke. However, a safe, reliable, and effective method for dynamic CBF evaluation is currently lacking in clinical practice. In this study, we developed a CBF monitoring system utilizing electromagnetic coupling sensing (ECS). This system detects variations in brain conductivity and dielectric constant by identifying the resonant frequency (RF) in an equivalent circuit containing both magnetic induction and electrical coupling. We evaluated the performance of the system using a self-made physical model of blood vessel pulsation to test pulsatile CBF. Additionally, we recruited 29 healthy volunteers to monitor cerebral oxygen (CO), cerebral blood flow velocity (CBFV) data and RF data before and after caffeine consumption. We analyzed RF and CBFV trends during immediate responses to abnormal intracranial blood supply, induced by changes in vascular stiffness, and compared them with CO data. Furthermore, we explored a method of dynamically assessing the overall level of CBF by leveraging image feature analysis. Experimental testing substantiates that this system provides a detection range and depth enhanced by three to four times compared to conventional electromagnetic detection techniques, thereby comprehensively covering the principal intracranial blood supply areas. And the system effectively captures CBF responses under different intravascular pressure stimulations. In healthy volunteers, as cerebral vascular stiffness increases and CO decreases due to caffeine intake, the RF pulsation amplitude diminishes progressively. Upon extraction and selection of image features, widely used machine learning algorithms exhibit commendable performance in classifying overall CBF levels. These results highlight that our proposed methodology, predicated on ECS and image feature analysis, enables the capture of immediate responses of abnormal intracranial blood supply triggered by alterations in vascular stiffness. Moreover, it provides an accurate diagnosis of the overall CBF level under varying physiological conditions.

## 1 Introduction

Stroke represents a significant global health threat, as evidenced by its high mortality and morbidity rates (Feigin et al., 2022). Numerous clinical guidelines recommend the control of vascular risk factors as the primary strategy for mitigating the impact of stroke (Suzuki et al., 2021). Clinical studies have shown that the immediate implementation of systemic thrombolysis and mechanical thrombectomy can significantly improve functional outcomes in cases of acute ischemic stroke (Fan et al., 2022; Nogueira et al., 2022; Yang et al., 2022). The primary goal of these interventions is to restore or preserve physiological blood flow in the brain. CBF can be measured both dynamically and statically. However, due to individual variations in pathogenic influences, extent of tissue damage, vascular resistance, and elasticity, the evaluation standards and range for optimal cerebral blood perfusion levels must be dynamically adjusted (Nie et al., 2023). As a result, dynamic CBF assessment is of greater clinical significance for guiding personalized management and treatment strategies, and improving the prognosis of stroke.

Currently, there is a lack of safe, reliable, and effective dynamic evaluation methods for CBF in clinical practice. CBF assessment primarily relies on computed tomography perfusion imaging (CTP) and diffusion-weighted imaging (DWI) (Martin et al., 2017). However, these imaging devices are typically bulky, which hinders bedside monitoring capabilities. Additionally, ischemic stroke (IS) patients in intensive care units may face challenges such as limited venous access and the risk of tracheal catheter displacement during intra-hospital transport to the imaging suite. Although clinical studies have demonstrated the potential of portable MRI in complex clinical care settings (Sheth et al., 2020), this device is not as sensitive to low-perfusion areas as professional perfusion imaging and is limited by a temporal window, preventing continuous tracking. The transcranial Doppler (TCD) technique allows for real-time evaluation of changes in cerebral hemodynamics and parenchymal structure through non-invasive measurement of CBF velocity (Tao et al., 2021). However, the TCD method is effective in tracking perfusion of larger vessels and is less effective in detecting perfusion in small to medium-sized vessels and microvessels (Gómez-Escalonilla et al., 2022). This limitation contributes to the ineffective clinical reperfusion observed post-vascular recanalization in IS patients. Electrical impedance tomography (EIT) offers non-invasive dynamic measurements of intracranial pathophysiological information. However, the low electrical conductivity of the skull impacts measurement accuracy (Wang et al., 2021). Near-infrared spectroscopy (NIRS) facilitates continuous non-invasive bedside monitoring of CBF by measuring changes in blood oxygenation and deoxygenation in blood vessels (Rivera et al., 2020). Despite these advantages, its detection depth is limited and accuracy is easily influenced by environmental factors. As such, NIRS is primarily suited for monitoring superficial tissue blood flow.

Electromagnetic coupling sensing (ECS) measures alterations in electrical conductivity and dielectric constant to procure pathophysiological information from biological tissues. Prior studies have proposed ECS as a promising innovation for the real-time bedside monitoring of cerebrovascular conditions such as traumatic brain injury, cerebral hemorrhage, and cerebral edema (Griffith et al., 2018; Alqadami et al., 2021; Li et al., 2021; Chen et al., 2022a; Chen and Kwai-Man, 2022; He et al., 2022). Pulsatile CBF, the changes in intracranial blood volume during cardiac systole and diastole in response to arterial blood pressure, provides insights into both immediate aberrations in intracranial blood supply and overall CBF levels. Consequently, it can offer comprehensive data for the dynamic assessment of intracranial blood supply. The formation of pulsatile CBF involves alterations in the relative volumes of major intracranial components like brain parenchyma, CBF, and cerebrospinal fluid. This process prompts swift responses in brain conductivity and dielectric constant. Employing magnetic induction phase shift signals, which indicate shifts in brain electrical conductivity, we separated pulsatile CBF (Zeng et al., 2022). And its primary frequency component is close to heart rate. In another experiment, inductive technology was used to observe pulsatile CBF in healthy volunteers during continuous inhalation of a certain concentration of CO_2_ (Zhang et al., 2023). Results indicated significant changes in time-domain and frequency-domain characteristics of the inductive signal correlated with the duration of CO_2_ inhalation. However, these investigations solely focused on changes in brain electrical conductivity in relation to pulsatile CBF, neglecting the variations in dielectric constant. More crucially, due to the lack of effective analytical methods and comparison with existing devices, the dependable association between ECS-based pulsatile CBF, the immediate response to intracranial blood supply abnormalities, and the overall level of CBF remains indeterminate.

In this study, we developed a CBF monitoring system utilizing ECS. This system detects variations in brain conductivity and dielectric constant by identifying the RF in an equivalent circuit containing both magnetic induction and electrical coupling. We evaluated the performance of the system using a self-made physical model of blood vessel pulsation to test pulsatile CBF. Additionally, we recruited 29 healthy volunteers to monitor CO, cerebral blood flow velocity (CBFV) data and RF data before and after caffeine consumption. We analyzed RF and CBFV trends during immediate responses to abnormal intracranial blood supply, induced by changes in vascular stiffness, and compared them with CO data. Furthermore, we explored a method of dynamically assessing the overall level of CBF by leveraging image feature analysis.

## 2 Materials and methods

### 2.1 Detection principle

**FIGURE 1:**
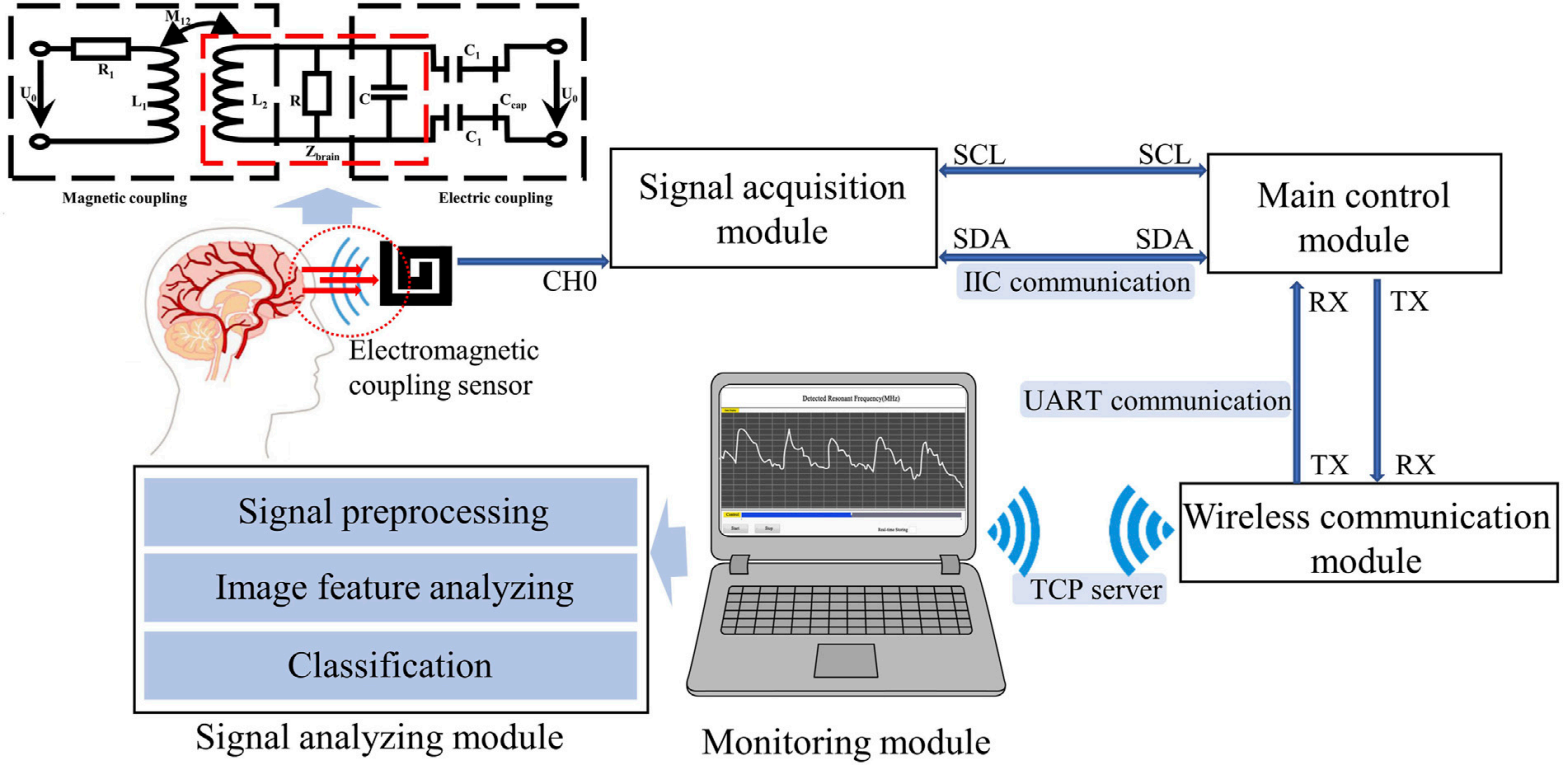
Overall design of the CBF detection device.

Within the near-field range, by applying an electromagnetic field to the skull and brain, both electrical coupling and magnetic induction can be achieved. The field distribution and corresponding circuit are illustrated in Figure 1. Magnetic induction causes a change in overall impedance $R_{ind}$ when the intracranial conductivity changes, while electrical coupling causes a change in coupling capacitance $C_{cap}$ when the intracranial dielectric constant changes. Based on the principle of electromagnetic coupling, Daniel Teichmann et al. derived the following relationship (Teichmann et al., 2013):

$$f_{osc}=\frac{1}{2\pi }\sqrt{\frac{1}{L\left(C+C_{cap}\right)}-{\left(\frac{R_{ind}}{2L}\right)}^{2}}$$

Where $f_{osc}$ is the RF, L is the no-load inductor, and C is the no-load capacitor.

Under the influence of arterial blood pressure, pulsatile CBF manifests a periodic response in tandem with cardiac systole and diastole, thereby maintaining dynamic balance in intracranial blood volume. This process triggers dynamic changes in the relative volumes of intracranial tissues, each having different electrical conductivities and dielectric constants. As per the above equation, the variations in electrical conductivity and dielectric constant of brain tissue can be ascertained by tracking changes in RF. Consequently, ECS can theoretically reflect information pertinent to pulsatile CBF.

### 2.2 CBF monitoring system

As illustrated in Figure 1, the electromagnetic coupling-based CBF monitoring system comprises an electromagnetic coupling sensor, signal acquisition module, main control module, wireless communication module, monitoring module and signal analyzing module. This system is designed to continuously and wirelessly monitor RF signals, which reflect the conductivity and permittivity of the measured target, and completes the dynamic assessment of the overall level of CBF through an algorithm combined with image features.

The electromagnetic coupling sensor was constructed by winding a coil around an electrode plate measuring 60 mm × 60 mm, fabricated from printed circuit board (PCB) material. The sensor boasts a double-layer coil winding, a track width of 4.5 mm, a turn spacing of 1.5 mm, and is comprised of copper-based material. The signal acquisition module, built with a capacitive data converter (FDC2214 EVM, TI) featuring an anti-electromagnetic interference architecture, was tasked with acquiring the detection results from the electromagnetic coupling sensor and calculating the RF. Communicating via the I2C protocol, the main control unit (STMF103C8T6) performs downsampling of the measurement findings at a rate of 20 Hz. The wireless communication module (ESP8266 ATK) retrieves data from the serial port and transmits them to the monitoring module using the TCP Server protocol. The monitoring module, developed with Labview software, is installed on a standard personal computer. It is responsible for displaying waveforms in real-time and storing monitoring data. The signal analysis module preprocesses the signal and uses a model that combines image feature analysis and machine learning to classify measured CBF signals at different levels. The entire system is powered by a 3.3 v lithium battery.

### 2.3 Performance evaluation experiments

**FIGURE 2:**
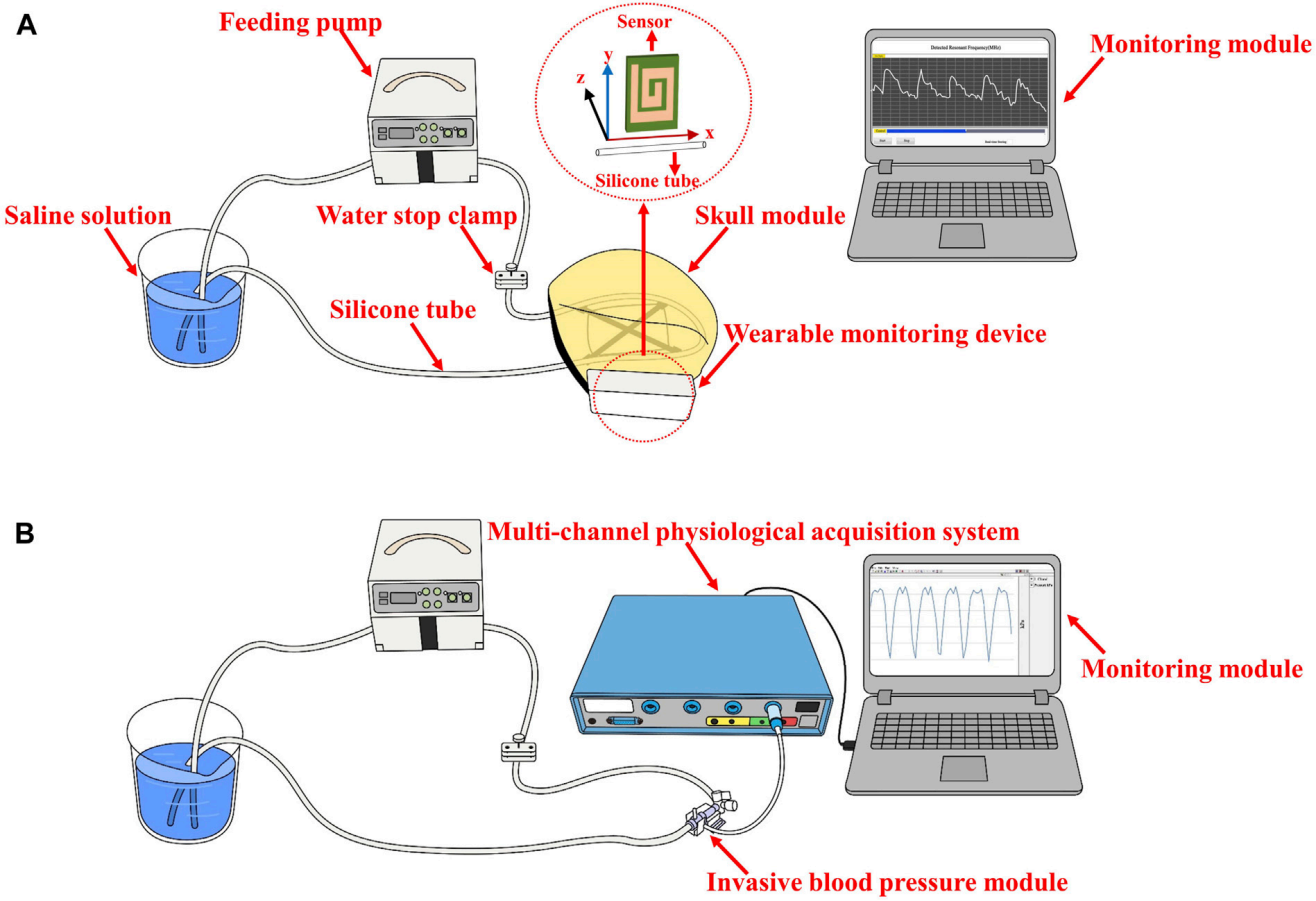
The setup of the performance evaluation experiments **(A)** The performance evaluation at various depths and range **(B)** CBF responses detection under different pressure excitations.

The performance evaluation experiments of the electromagnetic coupling-based CBF monitoring system were conducted using a simulated model of vascular pulsation. As shown in Figure 2, the model consists of a feed pump (ZNB-XY1; KellyMed, Beijing, China), a silicone tube (outer diameter = 5 mm, inner diameter = 3 mm), and a beaker filled with a saline solution (0.009 g/mL). The silicone tube was connected from the saline-filled beaker to the feed pump’s water inlet and back to the same beaker, simulating vascular pulsation. The feed pump’s squeeze frequency was set at 1 Hz, and the performance of the electromagnetic coupling-based CBF monitoring system was evaluated at various depths and ranges. For depth assessment, the silicone tube was displaced in the Oz direction from its initial position, ranging from 2 cm to 11 cm, advancing 1 cm at each step. For range evaluation, the silicone tube was shifted 4 cm in the Oy direction, with each step measuring 1 cm. To further examine the system’s ability to detect CBF responses under different pressure excitations, we installed a water stopper between the peristaltic pump’s outlet and the detection site. To mimic a more realistic detection environment, we incorporated a skull model and secured the system’s components (excluding the monitoring module) at the pterional region. The feed pump’s squeeze frequency was maintained at 2 Hz. The water stopper’s compression distance was modified (1.5 mm, 2 mm, 2.5 mm) to obtain CBF responses under diverse simulated intravascular pressure stimulations. Simultaneously, an invasive blood pressure measurement module (MMBPTSA20) was positioned identically and connected to a multi-parameter physiological monitor (RM6240XC) to measure the pressure signal.

### 2.4 CBF monitoring trials in health volunteers

In order to evaluate the feasibility of dynamic CBF monitoring via electromagnetic coupling sensing, a clinical trial was performed with 29 healthy volunteers (age range: 20–40 years). The work was approved by the Ethics Committee of the Southwest Hospital of Army Medical University (Chongqing, China). Each participant gave his or her informed permission. These individuals had no history of cardiovascular or cerebrovascular disorders and no implanted medical devices. The volunteers were split into experimental group 1 (*n* = 13), experimental group 2 (*n* = 13), and control group (*n* = 3). Participants in experimental group 1 and experimental group 2 consumed 200 mg of caffeine orally. This induced temporary alterations in the stiffness and elasticity of their cerebrovascular system, resulting in variations in their CBF. The photograph of simultaneous monitoring of RF and CO in health volunteers is provided in the Supplementary Material, during the trial in Experimental Group 1, participants remained seated in a restful state while the electromagnetic coupling sensor was secured to their right temple. RF measurements were captured continuously from before caffeine consumption to 30 min after caffeine consumption. The control group took the same measurements but did not consume caffeine. Concurrently, a CO monitor (MNIR-P100) was employed to gather the CO index as a reference. The control group followed an identical measurement method to the experimental group, except without the consumption of caffeine. Data for RF and CO was collected over a period of 5 min. To compare with standard CBFV data, we used transcranial Doppler (TCD) (EMS-9U) to measure cerebral blood flow parameters in the right middle artery in experimental group 2. CBFV data were measured before caffeine consumption and at 5-min intervals until 30 min after caffeine consumption.

The acquired signal data were subjected to a wavelet threshold denoising algorithm employing both soft and hard thresholds in order to filter out high-frequency noise, resulting in preprocessed signals. Subsequently, these preprocessed signals were utilized to analyze the dynamic variation in CBF following medication administration by using the change ratio of the amplitude (CRA).

### 2.5 Image feature analysis of CBF waveform

RF signals collected from the healthy volunteer experiment were converted into image data. Subsequently, image feature analysis was performed to evaluate the capability of the electromagnetic coupling system developed in this study to distinguish CBF levels under varying physiological states. The process of feature extraction and selection is illustrated in the Supplementary Material. Within a single physiological state for each participant, 16 data sets were obtained per minute, utilizing a fixed window of 300 RF signals and a step length of 60. Each data set was converted into a waveform graph, constructing the image dataset. A total of 336 images were generated for this study. Following this, image features were extracted from both local and overall aspects of the image, followed by dimension reduction through a selection process. Given the constraints of computational resources and time, the image size was adjusted to 100 × 100 pixels prior to feature extraction and selection, utilizing the bicubic interpolation method.

To extract overall image features, 7-dimensional and 16-dimensional features were calculated by employing Hu-Moment and the Gray Level Co-occurrence Matrix (GLCM) in multiple directions, respectively. Concurrently, the image’s Histogram of Oriented Gradient (HOG) and Local Binary Pattern (LBP) were utilized to acquire local image features. For the HOG, the image’s various gradient directions ranging from 0° to 360° were divided into 9 segments and further partitioned into several 16 × 16 blocks. Each block was split into four 8 × 8 cells. The gradient direction and magnitude for each pixel in every cell were calculated. Ultimately, the gradient histograms of multiple elements were amalgamated into high-dimensional vectors. For the LBP, the entire image was scanned in a 3 × 3 neighborhood to acquire texture information.

The distribution characteristics of the overall features extracted via Hu-Moment and GLCM were visualized and analyzed using line graphs for feature selection. The remaining global and local features were screened by computing the importance of random forest features. Features with an importance level higher than 0.5% were retained, while the rest were discarded. Following feature selection, a feature dataset was constructed and labeled with corresponding labels for subsequent training and testing of the classification model.

### 2.6 Overall CBF level classification

**FIGURE 3:**
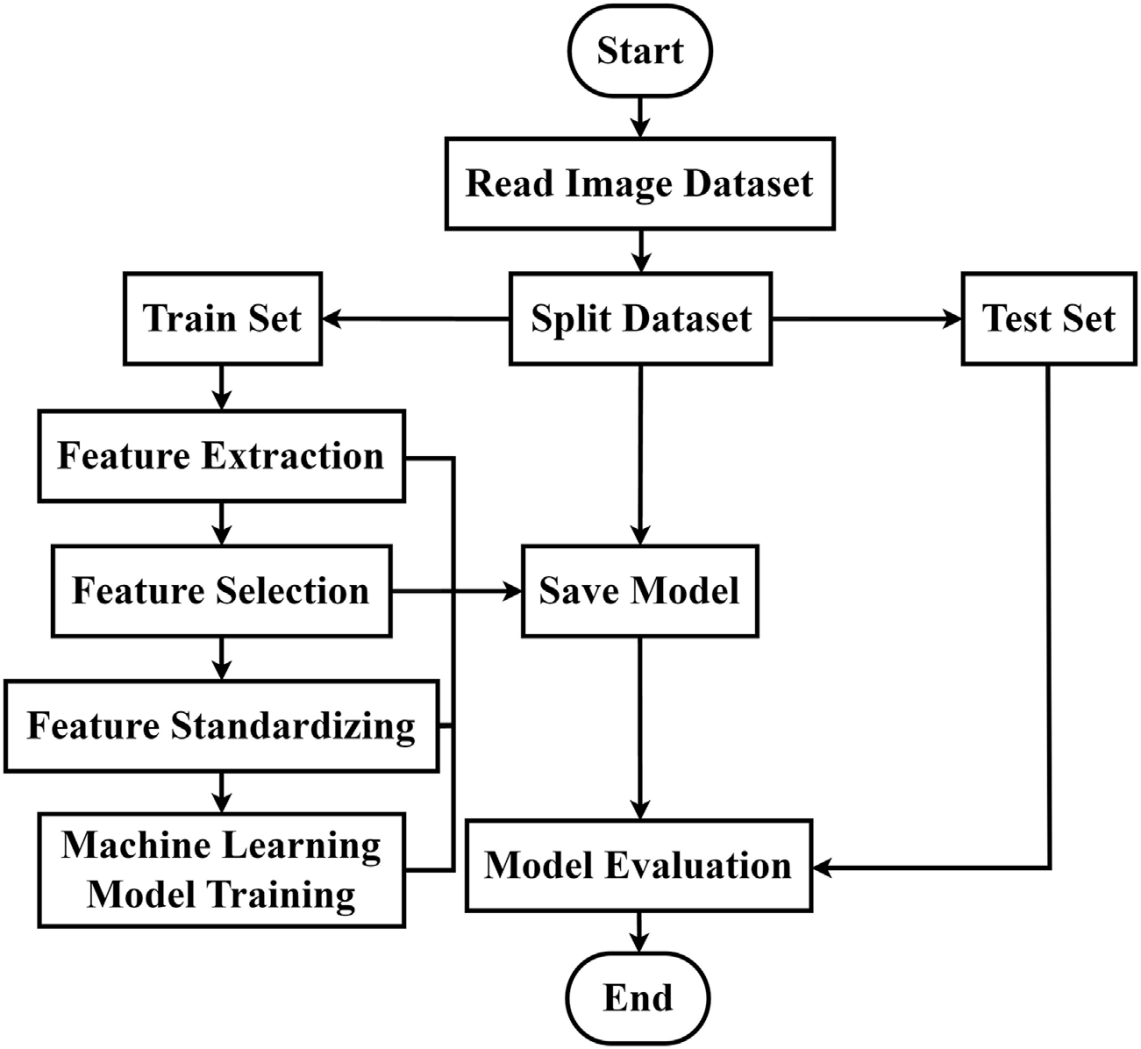
Classification algorithm flow of CBF levels in different physiological states.

Using machine learning methods, we constructed a diagnostic model for overall level of CBF before and after caffeine consumption, based on the feature dataset derived from image feature extraction and screening. The overall modeling process is depicted in Figure 3. The feature dataset was divided into a training set and a test set at a 4:1 ratio. To enhance the efficiency of the model training, the dataset was standardized. Random forest, Support Vector Machine (SVM), and K-Nearest Neighbor (KNN) algorithms were chosen for the training. A grid search was conducted using a 5-fold cross-validation method, iterating and adjusting parameters. The final output was the combination of parameters with the highest score. The tuning ranges of each model are shown in Table 1.

**TABLE 1:** Hyperparameter range settings for machine learning models.

	Parameter
KNN	n_neighbors = {3,4,5, … ,30} weights = {“uniform,” “distance”}
SVM	C = {0.01, 0.06,0.11, … ,5.01} gamma = {0.01, 0.11,0.21, … ,10.01}kernel = { “linear,” “rbf”}
Random Forest	n_estimators = {50,51,52, … ,201}
	max_features = { “sqrt,” “log2”}
	max_depth = {5,6,7,8,9,10, None}
	min_samples_split = {2,3,4,5}

In order to validate the performance of our model and identify the most effective algorithm, we leveraged a diverse array of metrics to scrutinize the classification efficacy of the three candidate machine learning algorithms. These metrics provided direct evaluation of the classification results, and included measures such as accuracy, recall, and the F1 score. In addition, we evaluated the specificity, or the true negative rate, of the classification model by utilizing Receiver Operating Characteristic (ROC) curves and computing the Area Under the Curve (AUC). Moreover, the interplay between accuracy and recall rates, key components of any classification problem, was rigorously analyzed through the deployment of Precision Recall (PR) curves and confusion matrices. This approach granted us an understanding of the model’s performance in terms of precision, recall, and the trade-off between these two critical metrics. By utilizing these comprehensive and nuanced evaluation methods, we were able to rigorously test and compare the performance of our candidate algorithms.

## 3 Results

**FIGURE 4:**
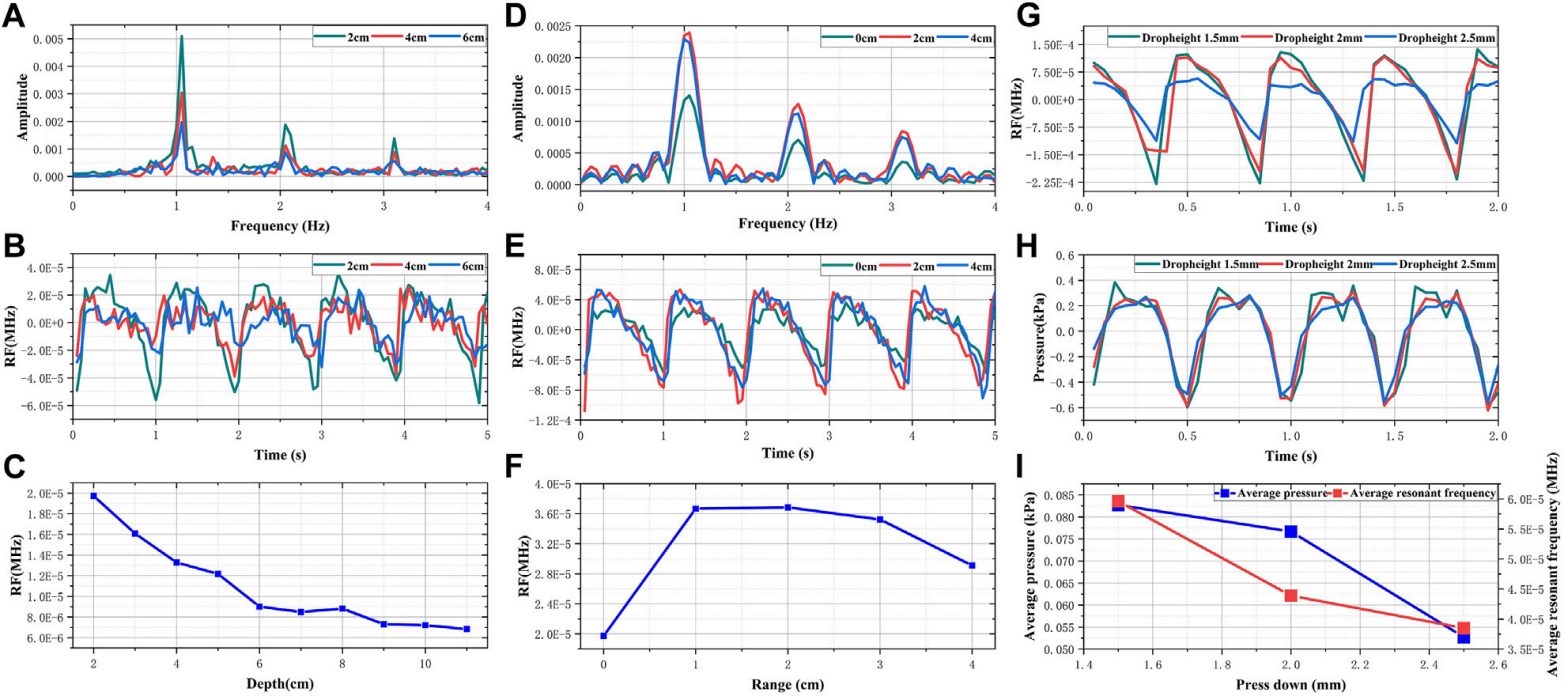
The result of performance evaluation experiments **(A)** Frequency domain RF signals of different depths **(B)** Time domain RF signals of different depths **(C)** The changes in sensitivity of RF signals with different depths **(D)** Frequency domain RF signals of different ranges **(E)** Time domain RF signals of different ranges **(F)** The changes in sensitivity of RF signals with different ranges **(G)** Comparison of RF signals in different dropheights **(H)** Comparison of pressure signals in different dropheights **(I)** Comparison of change trend between RF and simulated intravascular pressure.

When the feed pump’s extrusion frequency is held constant at 1 Hz, the monitoring results from the electromagnetic coupling system at varying depths are depicted in Figures 4A–C. We noted a periodic pulsation with pronounced peaks and troughs in the RF signal within the time domain as the detection depth was modified from 2 cm to 6 cm. Accompanying an increase in detection depth was a progressive diminution in the amplitude of these pulsations. In the frequency domain, the predominant frequency component of the RF exhibiting high amplitude were consistently found to be 1 Hz, thus corresponding to the feed pump’s squeeze frequency. At a depth of 8 cm, the average change in RF was observed to be on the order of 10–5. Referencing Daniel Teichmann’s study, the order of magnitude for observed values generally ranges from 10 to 3 to 10–5. Consequently, these findings suggest that the effective detection depth of the electromagnetic coupling system is approximately 8 cm. The monitoring results of the electromagnetic coupling system at different ranges are illustrated in Figures 4D–F. As the detection range shifted from 0 cm to 4 cm, the RF signal was found to pulsate at a frequency of 1Hz, and the average RF reading was on the order of 10–5. Given that the skull’s thickness is roughly 1.5 cm and the location of unilateral points is about 7–8 cm from the midline of the brain, it's notable that the results from the performance evaluation experiment demonstrate that the electromagnetic coupling-based CBF monitoring system can effectively encompass the blood supply area of the middle cerebral artery.

Figures 4G, H presents the simulated results of CBF responses incited by changes in intravascular pressure. As the descending height increases, both the amplitude of the invasive blood pressure wave and the periodic fluctuation range of RF are observed to decrease. As illustrated in Figure 4I, with the pressing distance escalating from 1.5 mm to 2.5 mm, the pressure within the tube diminishes from 0.0827 kPa to 0.05274 kPa. Concurrently, the average RF declined from 5.96 × 10^−5^ MHz to 3.86 × 10^−5^ MHz. When the extrusion frequency of the feed pump remains unchanged, the pressure within the tube is directly proportional to the simulated blood flow per unit time (Mohammed et al., 2019; Barvik et al., 2023). Theoretically, the periodic pulsation range and average value of RF should adhere to the same trend as the pressure within the tube. Hence, these results substantiate the assertion that electromagnetic coupling-based systems exhibit the potential to discern CBF responses under varying pressure stimulations.

**FIGURE 5:**
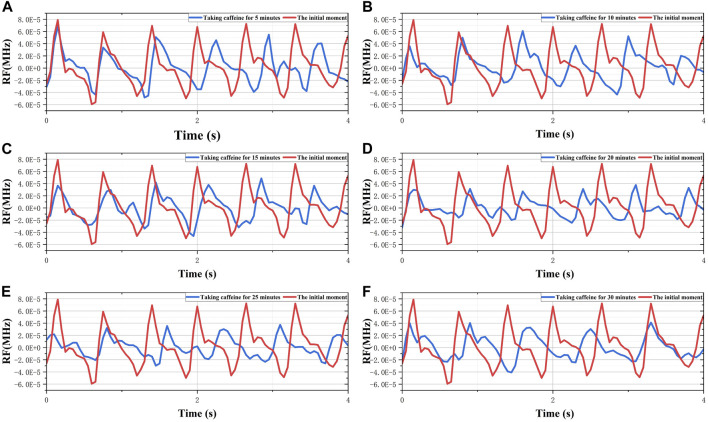
The 30-min monitoring results of RF in the No.1 healthy volunteer after taking caffeine. **(A)** Comparison of RF between the initial moment and taking caffeine for 5 min. **(B)** Comparison of RF between the initial moment and taking caffeine for 10 min. **(C)** Comparison of RF between the initial moment and taking caffeine for 15 min. **(D)** Comparison of RF between the initial moment and taking caffeine for 20 min. **(E)** Comparison of RF between the initial moment and taking caffeine for 25 min. **(F)** Comparison of RF between the initial moment and taking caffeine for 30 min.

**FIGURE 6:**
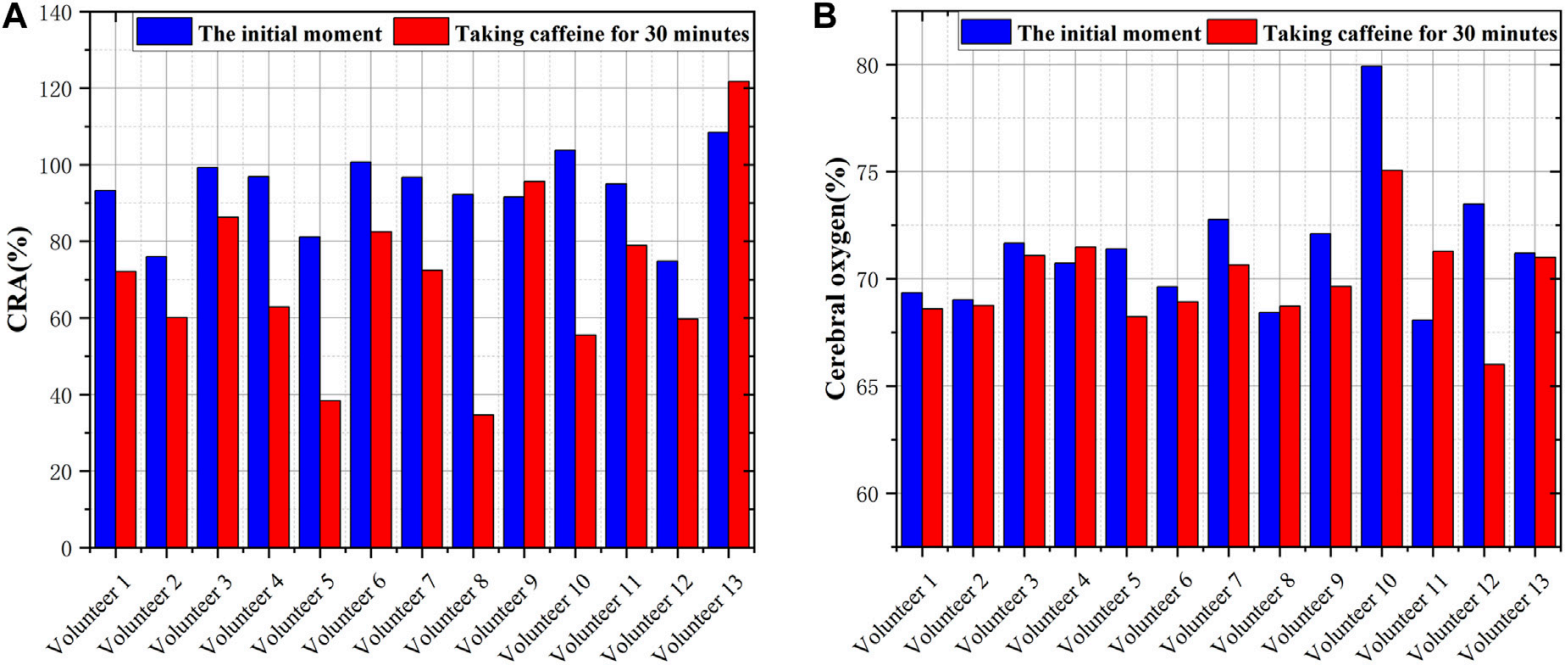
Comparison of CRA and CO before and after consuming caffeine **(A)** The CRA in volunteers at different states **(B)** The CO in volunteers at different states.

**FIGURE 7:**
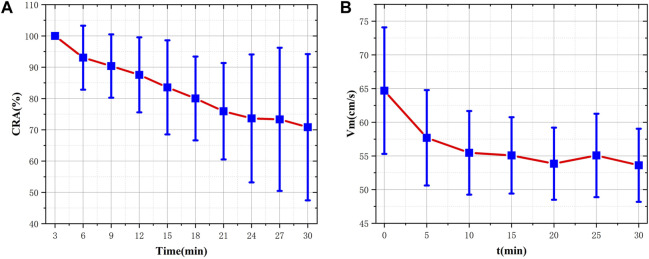
Comparison of CRA and VM before and after consuming caffeine **(A)** The distribution and fluctuating pattern of CRA over time **(B)** The distribution and fluctuating pattern of Vm over time.

The pretreated RF monitoring results for Volunteer 1 are displayed in Figure 5. The RF signal exhibits periodic pulsations with pronounced peaks and troughs. This signal includes a primary wave caused by blood vessel constriction and a secondary wave caused by blood vessel rebound (Chen et al., 2022b). The blue lines in Figures 5A–F present the changes in RF pulsations 30 min post-caffeine consumption, with observations recorded at five-minute interval, and the red lines show RF signals before caffeine consumption. The ingestion of caffeine can trigger transient disturbances in CBF (Liu et al., 2021). Over time, the amplitude of the RF pulsations progressively diminishes, with the most significant decline apparent 25 min after caffeine consumption. These results suggest a potential relationship between the change in RF pulsation amplitude and CBF disturbance induced by caffeine consumption. The increased arterial stiffness and diminished elasticity resulting from caffeine ingestion leads to a decrease in CBF. The CO level is proportional to CBF. The CRA relative to normal, both 5 min and 30 min post-caffeine ingestion, for all volunteers is depicted in Figure 6A. Among the 13 subjects, only two exhibited an increase in the CRA, while the remainder demonstrated a downward trend to varying degrees. As shown in Figure 6B, for most healthy volunteers, the initial CO index post-caffeine consumption was greater than the CO index at 30 min, suggesting that caffeine intake reduced CO levels. Systolic velocity (Vs), diastolic velocity (Vd) and mean cerebral blood flow velocity (Vm) in experimental group 2 showed similar changes. Figures 7A, B show the trend graphs of Vm measured by TCD and CRA of RF signal after oral caffeine consumption, respectively. After caffeine consumption, there was an overall decrease in Vm over time, which is consistent with the trend in CRA. The Spearman rank correlation coefficient between Vm and CRA was 0.8986, and the *p*-value was 0.0148, indicating that the correlation was significant. These results confirm that the electromagnetic coupling-based CBF monitoring system can effectively reflect the immediate response to abnormal intracranial blood supply provoked by changes in vascular stiffness post-caffeine consumption in healthy volunteers.

**FIGURE 8:**
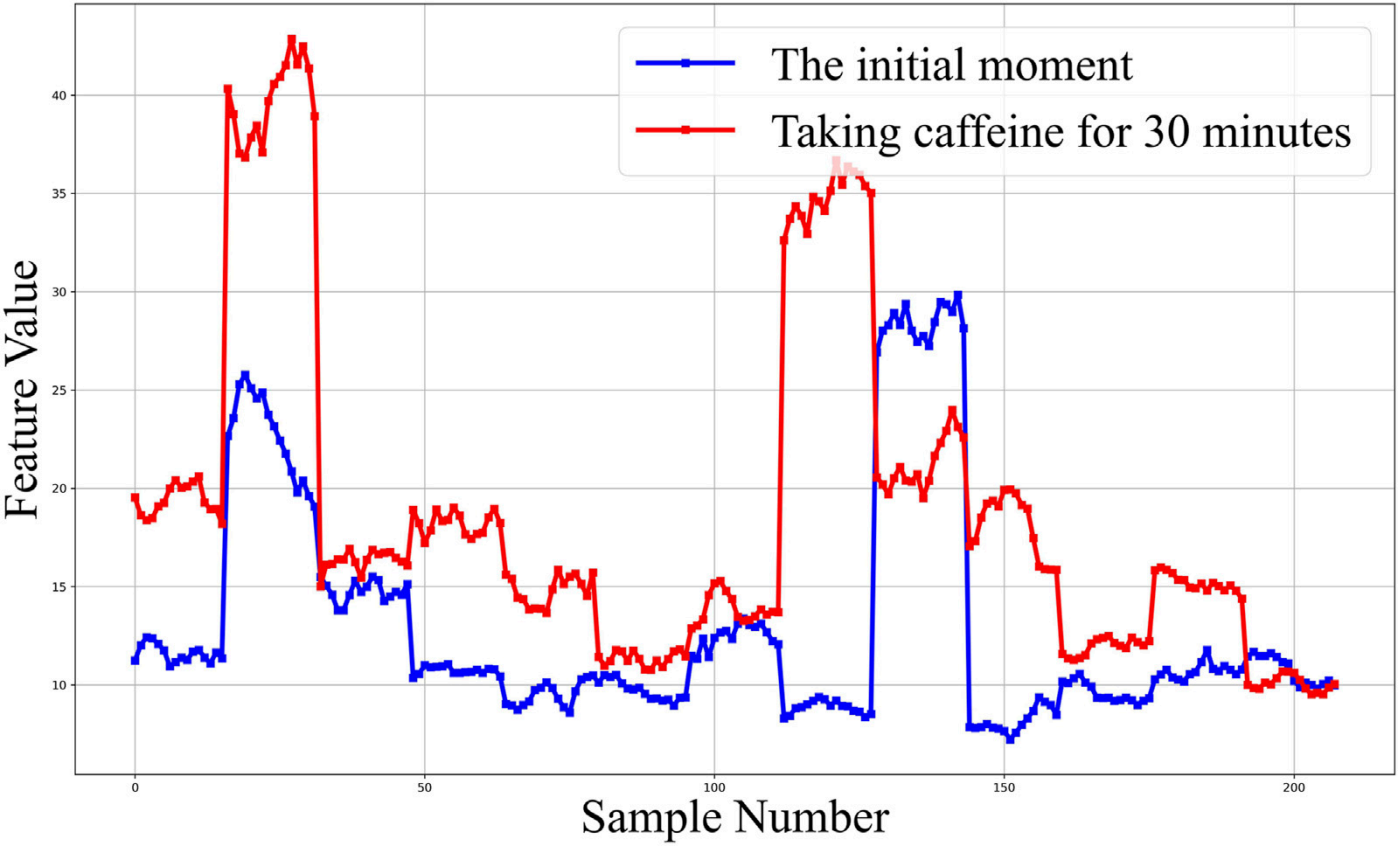
Contrast feature analysis diagram of 0° gray co-occurrence matrix.

We extracted a total of 14,379 dimensions of CBF waveform features. Among these, the global features comprised 23 dimensions and the local features encompassed 14,356 dimensions. For the global features, we conducted an analysis for the 0° GLCM illustrated in Figure 8. It can be discerned that the feature values for the majority of volunteers showed a significant increase post-caffeine ingestion. Furthermore, we performed analyses on other GLCM features and found that approximately 70% of the contrast and entropy values in four directions of GLCM exhibited a notable upward trend. Conversely, the energy and loss moment demonstrated a corresponding downward trend. The comparison graphs for the remaining features are provided in the Supplementary Material. Subsequently, we decided to retain only the GLCM features and conduct feature selection on Hu-moments and local features based on their feature importance. During this process, we computed feature importance and discarded those with an importance value of less than 0.5%. The final feature retention results are presented in Table 2. We retained 9 dimensional HOG features and 9 dimensional LBP features. Combined with the previous GLCM features, a total of 34 dimensional features were preserved for subsequent studies. The Hu-moment features, due to their low importance and irregular distribution, were discarded.

**TABLE 2:** Feature screening.

	Total feature dimension	Retained feature dimension	Whether to discard
GLCM	16	16	No
HOG	4,356	9	No
HuMoment	7	0	Yes
LBP	10,000	9	No

**FIGURE 9:**
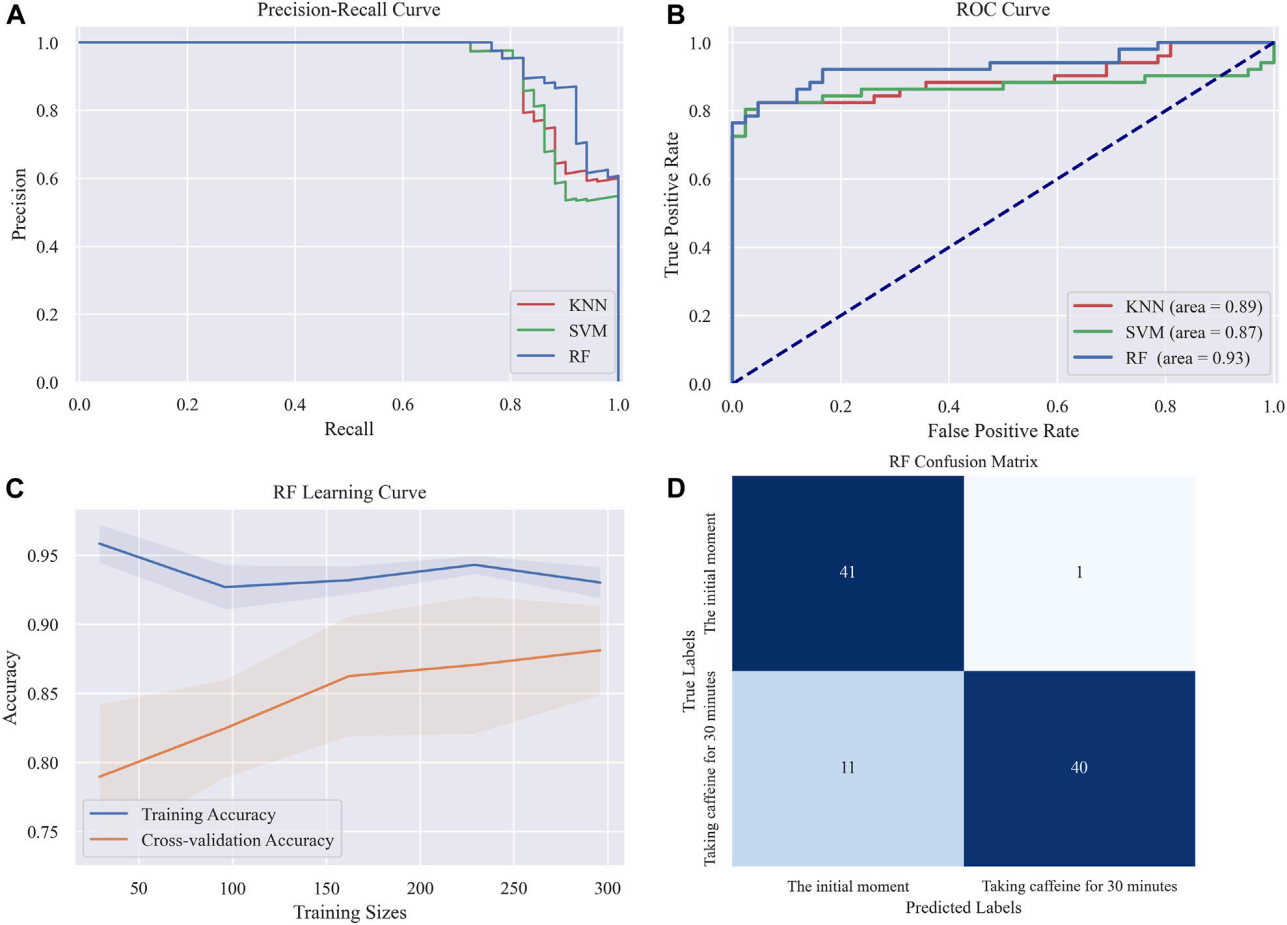
Graphical interpretations of the classification performance in the three machine learning models **(A)** PR curve **(B)** ROC curve **(C)** Random forest Learning curve **(D)** Random forest confusion matrix.

We trained and tested three distinct machine learning models, namely, Random Forest, KNN, and SVM, using the retained features. As shown in Table 3, the Random Forest model exhibits the best performance across all measured parameters, achieving an accuracy of 87.09%, an F1 score of 0.8867, and an AUC value of 0.93. Figure 9 presents the graphical interpretations of the performance of the three machine learning models, including the PR curve, ROC curve, confusion matrix, and learning curve. Analyses of these graphs attest to the high performance of the Random Forest model in terms of both precision and recall, resulting in a commendable F1 score. The learning curve shows that the accuracy of the test set steadily increases with the expansion of the dataset and sustains a satisfactory level, indicating that there is no underfitting or overfitting within the classification model. Overall, the Random Forest model presents superior performance for this particular task. These results provide preliminary evidence that our method can accurately classify different changes in abnormal intracranial blood supply caused by changes in blood vessel stiffness. Additionally, it provides a method for diagnosing overall CBF levels under different physiological conditions.

**TABLE 3:** Classification test results of different models.

	SVM	KNN	Random forest
F1	0.8737	0.8679	0.8867
Accuracy	0.8602	0.8494	0.8709
AUC	0.87	0.89	0.93

## 4 Discussion

The dynamic assessment of CBF carries significant clinical value, as it provides crucial guidance in determining personalized management and treatment strategies, ultimately leading to improved stroke prognoses. Currently, the lack of a safe, reliable, and effective bedside method for evaluating CBF dynamics continues to result in a considerable portion of IS patients experiencing ineffective CBF reperfusion, despite vascular recanalization. Notably, approximately 50% of IS patients, who underwent successful vascular recanalization within 24 h as suggested by a superior modified Thrombolysis in Cerebral Infarction (mTICI) assessment, failed to demonstrate a favorable functional outcome after 90 days (Jovin et al., 2022). This study aims to extract pulsatile CBF by observing changes in cerebral electrical conductivity and the dielectric constant during cardiac systolic and diastolic periods. These changes serve to dynamically reflect the immediate response of intracranial blood supply abnormalities. By leveraging image feature analysis and machine learning modeling, it provides a method for diagnosing overall CBF levels under different physiological conditions.

The large size and low temporal resolution of CT, MRI, and other imaging devices, coupled with the high price of these devices, place a heavy financial burden on individuals. The wearable cerebral blood flow monitoring device established in this study has significant advantages of small size and low cost. The characteristics of lithium battery power supply and wireless communication enable the device to carry out real-time and continuous monitoring of CBF on mobile terminal or PC, which has high operability and portability, does not require too much operation skills, and has more application scenarios. In contrast to traditional methodologies that solely detect changes in either electrical conductivity or the dielectric constant, a more reliable extraction of pulsatile CBF can be achieved through a combined detection of electrical coupling and magnetic induction in the near-field range. The electric coupling equivalent circuit, predominantly used for detecting changes in the dielectric constant of the subject, exhibits high sensitivity, albeit with compromised penetration capabilities (Maharani et al., 2020). Conversely, magnetic induction, capable of penetrating the skull, allows the measurement of brain tissue’s electrical conductivity by gauging the disturbances of induced eddy currents within the intracranial region. However, given the relatively low electrical conductivity of biological tissues, the ensuing interference is relatively weak, leading to suboptimal detection sensitivity (Oziel et al., 2019). Exploiting the inherent non-interference between electric fields and magnetic fields, this study presents an ECS, which incorporates a sensor constructed via coil winding on the electrically coupled substrate, thus enabling the complementary use of electric coupling and magnetic induction. To assess the performance of the proposed system, we conducted range and depth tests on a self-made physical model of blood vessel pulsation. The experimental results indicate that RF changes of the order of 10–5 can still be detected at a vertical distance of 8 cm and a lateral displacement of 4 cm, with pronounced periodic pulsations identifiable between peaks and troughs. Compared to existing induction-based CBF assessment system, the effective detection range and depth of our proposed system are enhanced by a factor of three to four (Zeng et al., 2022; Zhang et al., 2023).

The electromagnetic coupling-based monitoring system demonstrates the capability to discern changes in blood flow volume resulting from simulated internal pressure within the vessel. This indicates that the system is capable of detecting CBF responses to spontaneous excitation of ABP. When ABP deviates within a specified range, the cerebrovascular system safeguards a stable CBF state through active dilation and contraction, constituting cerebrovascular reactivity (CVR). Numerous risk factors associated with cerebrovascular disease commonly engender a diminishment of CVR and augmented blood pressure variability (Gomez et al., 2021). The cumulative effect of these factors can precipitate cerebral blood perfusion deficiency or inflict mechanical overload on vascular walls, thereby hastening the progression of cerebrovascular diseases (de Heus et al., 2020). Presently, clinical CVR testing largely depends on the reaction of CBF to standard ABP changes induced by pharmacological, venous, carotid compression, and thigh cuff methods (Petkus et al., 2019). Nevertheless, the routine stimulation of blood pressure alterations entails potential risks and its utilization in intensive care is circumscribed. Detecting CBF responses to spontaneous excitation of ABP could effectively resolve this issue. In instances of unwavering vibration frequency, the diameter of the simulated vascular system escalates with the increase in internal pressure. Although this is incongruous with the physiological mechanism of vasoconstriction elicited by an elevated ABP, the trend of RF pulsation amplitude harmonizes with simulated intravascular pressure. The frequency of CBF arising from spontaneous ABP oscillation approximates heart rate and exhibits individual variability. In performance evaluation experiments, the vibration frequency of simulated blood vessels aligns with the primary frequency domain component of RF. During trials involving healthy volunteers, the low-frequency components of RF were observed to vary within the normal heart rate. Therefore, this study lays the groundwork for the formulation of novel methods for CVR evaluation.

The pulsatile CBF extracted based on ECS can dynamically reflect the immediate response of intracranial blood supply abnormalities caused by changes in vascular stiffness. When compared with continuous CO2 inhalation or breath-holding techniques, the aberrant intracranial blood supply instigated by oral caffeine administration aligns more closely with the pathophysiological progression of cerebral ischemia (Wardlaw et al., 2019). Oral caffeine consumption can induce alterations in vascular stiffness, which subsequently lead to hypervolemic blood flow in intracranial microvasculature. The cerebral sensitivity to heightened pulsatile blood flow initiates morphological transformations in intracranial microvessels, such as diminished internal diameter and reduced systolic and diastolic volumes (Gil et al., 2022). As the remodeling and stiffness of intracranial microvasculature increase, deficiencies in CBF due to ischemia and hypoxia become evident (Freitas-Andrade et al., 2020). The readily accessible methods for assessing CBF are CBFV measured by TCD and CO measured by NIRS. Considering the pathophysiological changes in intracranial microvessels are the key contributors to insufficient CBF supply post-caffeine consumption, we employed CO and CBFV as a reference to analyze and compare the RF measurement results in this study. In comparison to the initial state, the CO index of healthy volunteers demonstrated a substantial decline 30 min post-caffeine ingestion. However, the extent of changes in CO was not uniform, which may be attributable to individual variations in caffeine tolerance. Notably, the CRA and CBFV exhibited a downward trend within 30 min of caffeine consumption. As the magnitude of RF symbolizes the intensity of pulsatile CBF, these results suggest that the amplitude alterations in the RF signal mirror the immediate abnormal responses of intracranial blood supply triggered by caffeine.

Combined with image feature analysis and machine learning modeling, ECS can achieve dynamic assessment of the overall level of CBF. There is a close correlation between CO and CBF, with cerebral hypoxia typically lagging behind CBF deficiency. As such, the significant drop in CO levels observed 30 min after oral caffeine ingestion in healthy volunteers suggests a decrease in overall intracranial blood volume. For this study, we leveraged image feature analysis to generate a dataset discerning the overall level of intracranial blood volume. Compared to other feature extraction algorithms, image features offer distinct advantages in terms of interpretability, richness of information, and robustness. We processed local and global features of the image separately. Global features primarily reflect information such as texture thickness, randomness, area, and size (Tian et al., 2020), while local features detail the shape and gradient of specific image details (Zhang et al., 2021). The combination of global and local features elucidates the irregularity, amplitude size, area, and smoothing degree of the RF waveform, details that are intrinsically linked to CBF’s intensity, velocity, periodic change, and volume size. By extracting image features and selecting features based on importance computation and visualization, we can amass a feature dataset that holistically reflects overall changes in CBF levels. Leveraging this dataset, we employed machine learning to construct a classification model with high accuracy, facilitating dynamic assessment for the overall CBF level. The three principal machine learning algorithms currently used—Random Forest, KNN, and SVM—have different training methods. Utilizing these three algorithms for research allows us to analyze image feature information from various perspectives and enhance the universality and reliability of our findings. According to the classification results, the accuracy of different models varies. This discrepancy stems from the distinct performance of various features within and between groups, with different models amplifying this difference.

However, this is a preliminary study. The instantaneous response of CBF abnormalities under the combined stimulation of vascular pressure and stiffness has not been assessed by ECS due to limitations in the simulated model of vascular pulsation. The classification performance of overall CBF levels is constrained by the lack of complex texture image features. In next research phase, our team will develop a physically simulated model approximating actual vascular pulsations and investigate the impact of multiple parameters (CBF, ABP, and vessel diameter) on RF. Additionally, we will explore various data encoding methods to transform one-dimensional data into two-dimensional images, aiming to obtain more intricate texture features and enhance classification performance.

## 5 Conclusion

This study presents a dynamic assessment methodology for CBF by integrating electromagnetic coupling sensing and image feature analysis. The CBF monitoring system, based on electromagnetic coupling sensing, detects changes in brain conductivity and dielectric constant by employing RF in an equivalent circuit that includes magnetic induction and electrical coupling. Experimental testing substantiates that this system provides a detection range and depth enhanced by three to four times compared to conventional electromagnetic detection techniques, thereby comprehensively covering the principal intracranial blood supply areas. The system effectively captures CBF responses under different intravascular pressure stimulations. In healthy volunteers, as cerebral vascular stiffness increases and CO decreases due to caffeine intake, the RF pulsation amplitude diminishes progressively. Upon extraction and selection of image features, widely used machine learning algorithms exhibit commendable performance in classifying overall CBF levels before and after caffeine consumption. These results highlight that our proposed methodology, predicated on ECS and image feature analysis, enables the capture of immediate responses of abnormal intracranial blood supply triggered by alterations in vascular stiffness. Moreover, it provides an accurate diagnosis of the overall CBF level under varying physiological conditions.

## Data Availability

The raw data supporting the conclusion of this article will be made available by the authors, without undue reservation.

## Supplementary material

SUPPLEMENTARY FIGURE S1 The simultaneous monitoring of RF and CO in health volunteers.

SUPPLEMENTARY FIGURE S2 The flow chart of image feature extraction and analysis.

SUPPLEMENTARY FIGURE S3 Contrast feature analysis diagram of 0° gray co-occurrence matrix.

SUPPLEMENTARY FIGURE S4 Entropy feature analysis diagram of 0° gray co-occurrence matrix.

SUPPLEMENTARY FIGURE S5 Energy feature analysis diagram of 0° gray co-occurrence matrix.

SUPPLEMENTARY FIGURE S6 Deficit moment feature analysis diagram of 0° gray co-occurrence matrix.

SUPPLEMENTARY FIGURE S7 Contrast feature analysis diagram of 90° gray co-occurrence matrix.

SUPPLEMENTARY FIGURE S8 Entropy feature analysis diagram of 90° gray co-occurrence matrix.

SUPPLEMENTARY FIGURE S9 Energy feature analysis diagram of 90° gray co-occurrence matrix.

SUPPLEMENTARY FIGURE S10 Deficit moment feature analysis diagram of 90° gray co-occurrence matrix.

SUPPLEMENTARY FIGURE S11 Contrast feature analysis diagram of 45° gray co-occurrence matrix.

SUPPLEMENTARY FIGURE S12 Entropy feature analysis diagram of 45° gray co-occurrence matrix.

SUPPLEMENTARY FIGURE S13 Energy feature analysis diagram of 45° gray co-occurrence matrix.

SUPPLEMENTARY FIGURE S14 Deficit moment feature analysis diagram of 45° gray co-occurrence matrix.

SUPPLEMENTARY FIGURE S15 Contrast feature analysis diagram of 135° gray co-occurrence matrix.

SUPPLEMENTARY FIGURE S16 Entropy feature analysis diagram of 135° gray co-occurrence matrix.

SUPPLEMENTARY FIGURE S17 Energy feature analysis diagram of 135° gray co-occurrence matrix.

SUPPLEMENTARY FIGURE S18 Deficit moment feature analysis diagram of 135° gray co-occurrence matrix.

SUPPLEMENTARY FIGURE S19 h0 feature analysis diagram of Hu-Moment.

SUPPLEMENTARY FIGURE S20 h1 feature analysis diagram of Hu-Moment.

SUPPLEMENTARY FIGURE S21 h2 feature analysis diagram of Hu-Moment.

SUPPLEMENTARY FIGURE S22 h3 feature analysis diagram of Hu-Moment.

SUPPLEMENTARY FIGURE S23 h4 feature analysis diagram of Hu-Moment.

SUPPLEMENTARY FIGURE S24 h5 feature analysis diagram of Hu-Moment.

SUPPLEMENTARY FIGURE S25 h6 feature analysis diagram of Hu-Moment.
