# Supplementary figures and images for: Dynamic cerebral blood flow assessment based on electromagnetic coupling sensing and image feature analysis
Source: Front Bioeng Biotechnol. 2024 Feb 21;12:1276795. doi: 10.3389/fbioe.2024.1276795 (PMC10915240; doi:10.3389/fbioe.2024.1276795)

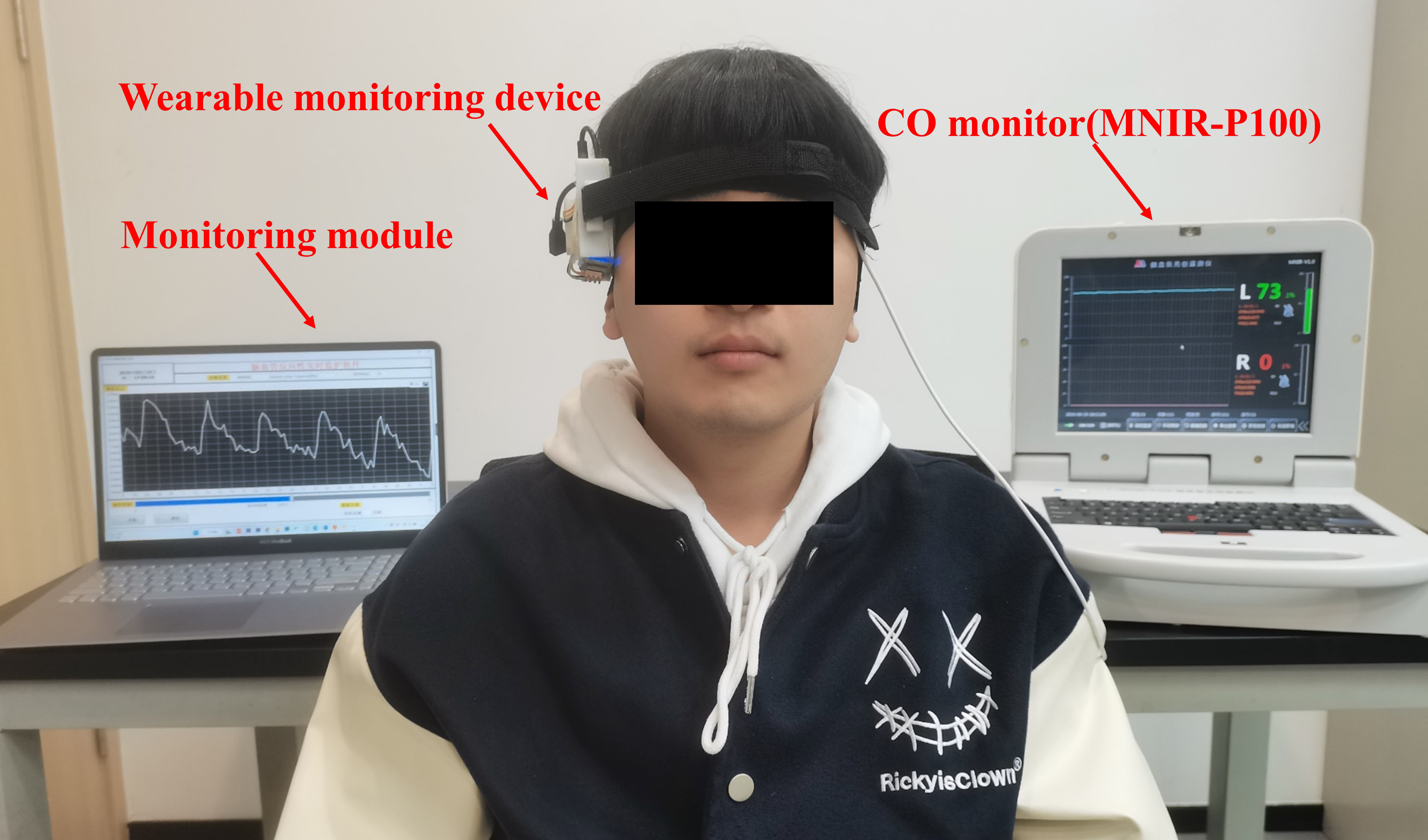

Supplement: Supplementary file 1 [file DataSheet1.ZIP › Suppl.image 1.jpg]

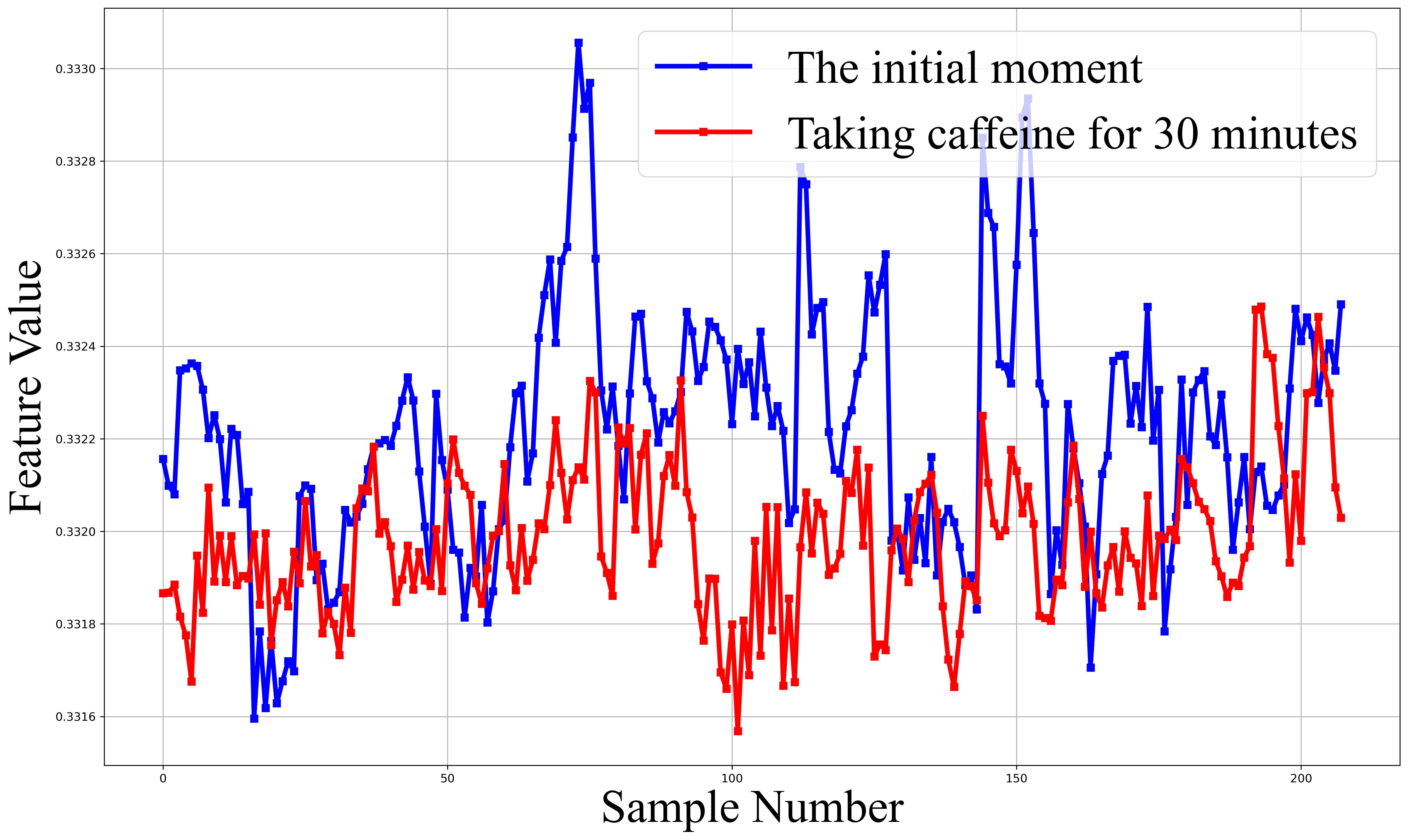

Supplement: Supplementary file 1 [file DataSheet1.ZIP › Suppl.image 10.jpg]

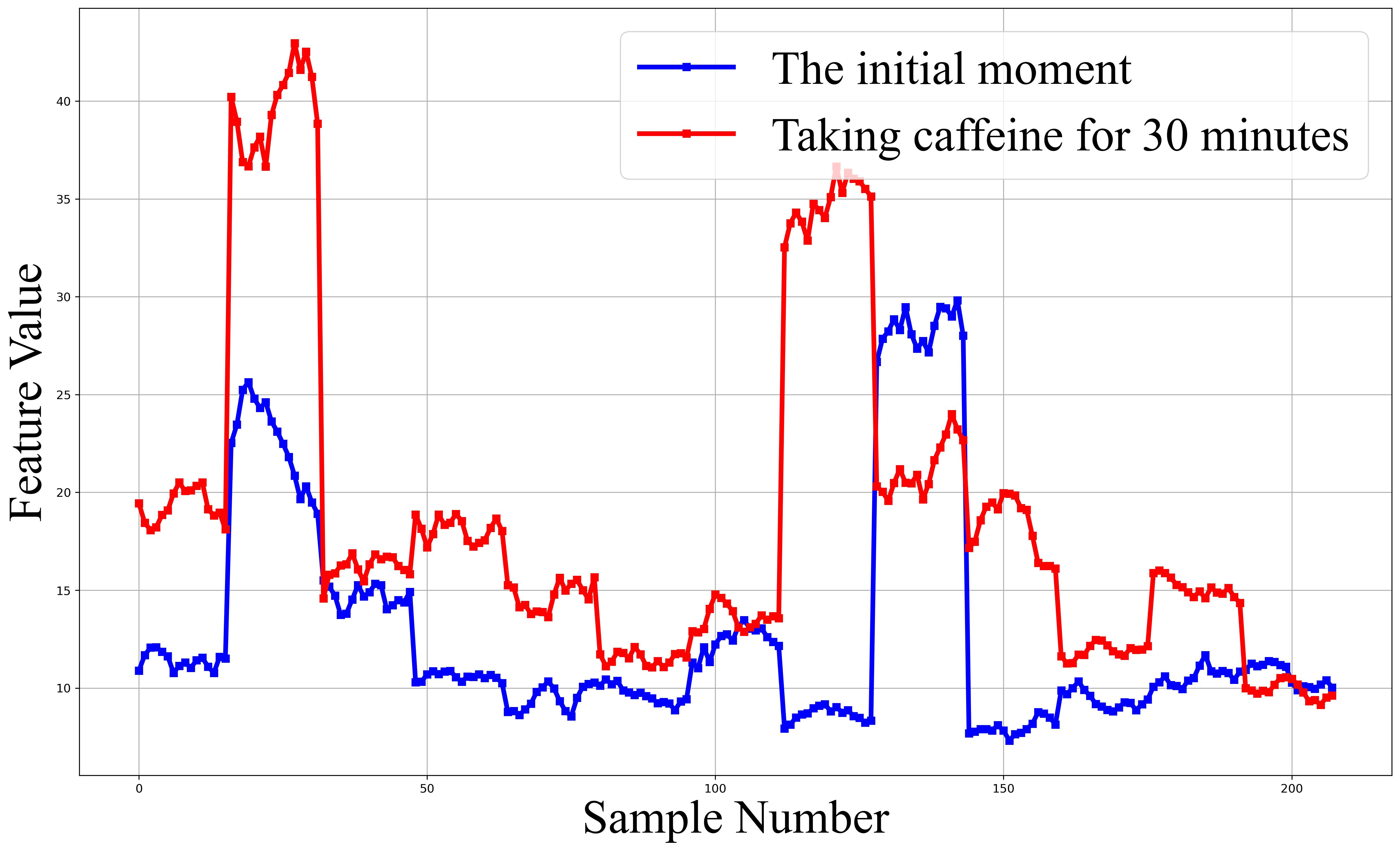

Supplement: Supplementary file 1 [file DataSheet1.ZIP › Suppl.image 11.jpg]

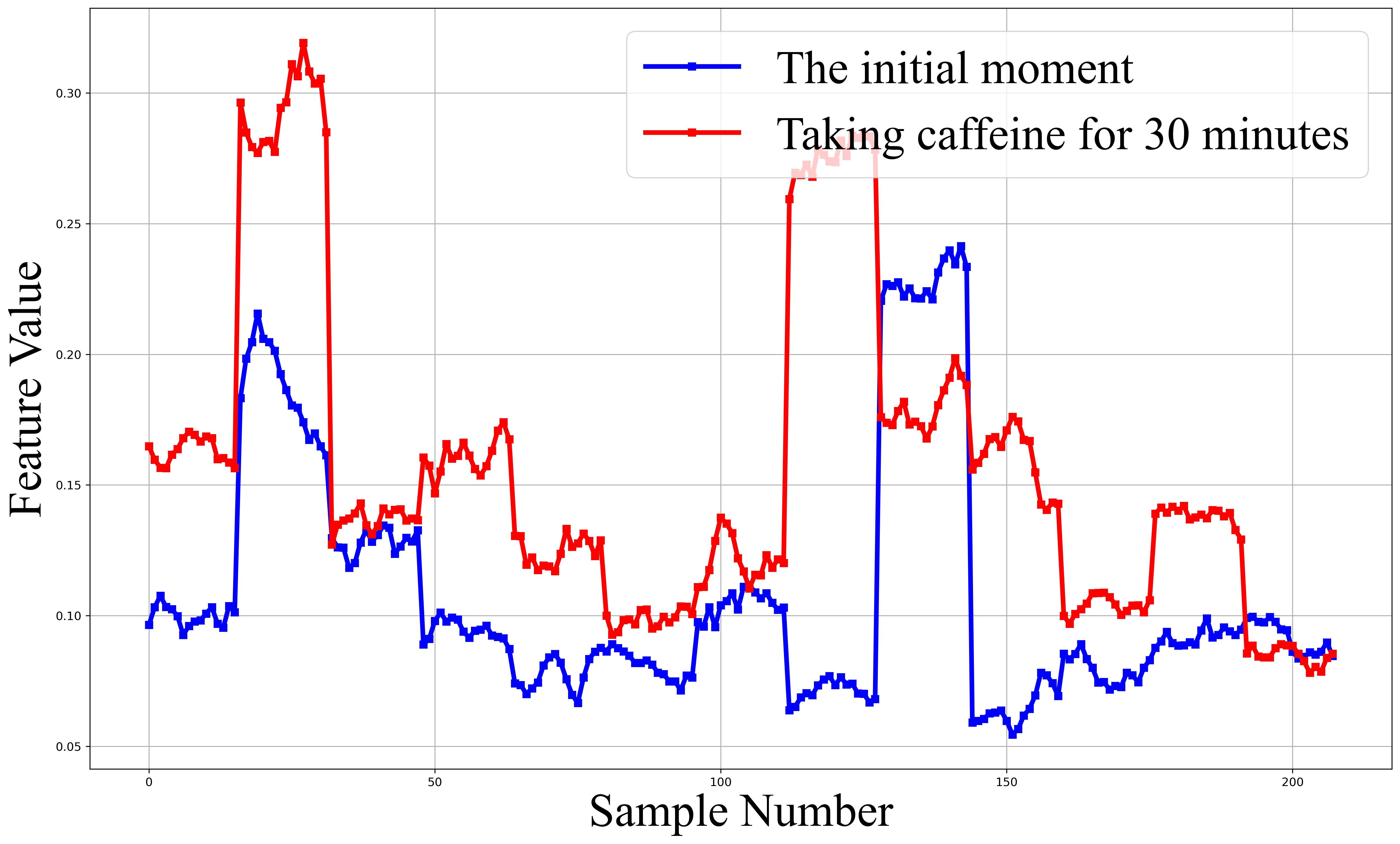

Supplement: Supplementary file 1 [file DataSheet1.ZIP › Suppl.image 12.jpg]

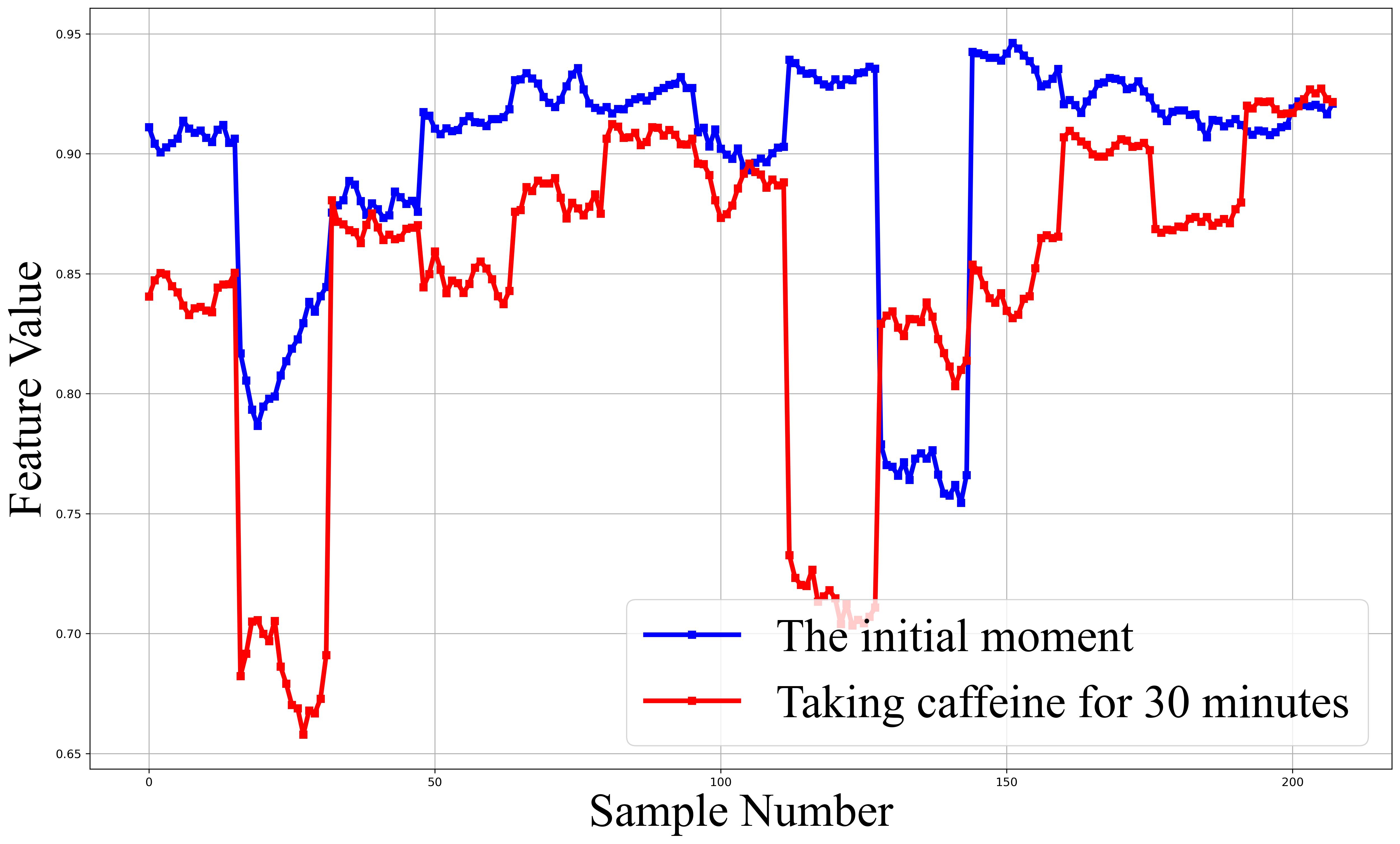

Supplement: Supplementary file 1 [file DataSheet1.ZIP › Suppl.image 13.jpg]

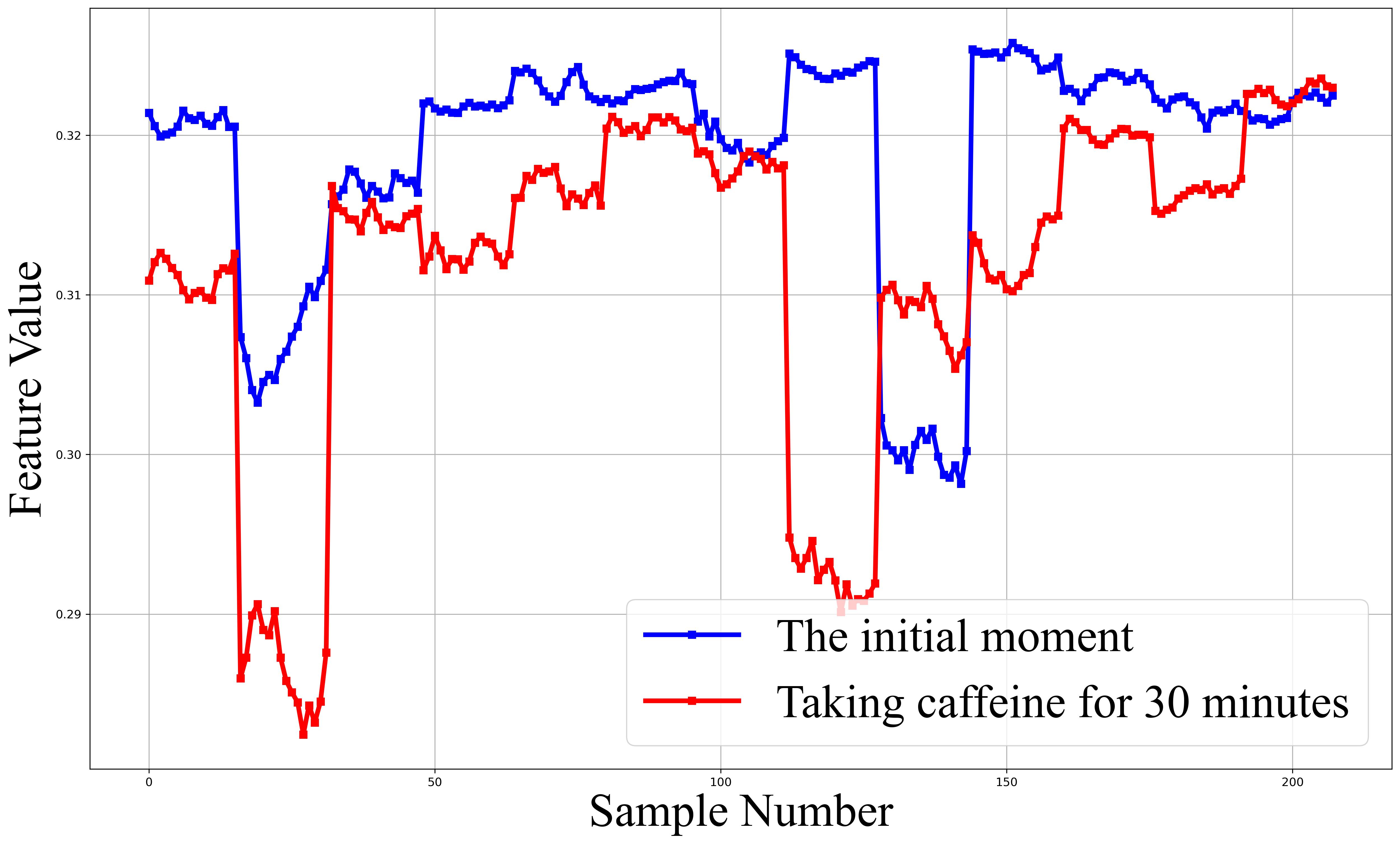

Supplement: Supplementary file 1 [file DataSheet1.ZIP › Suppl.image 14.jpg]

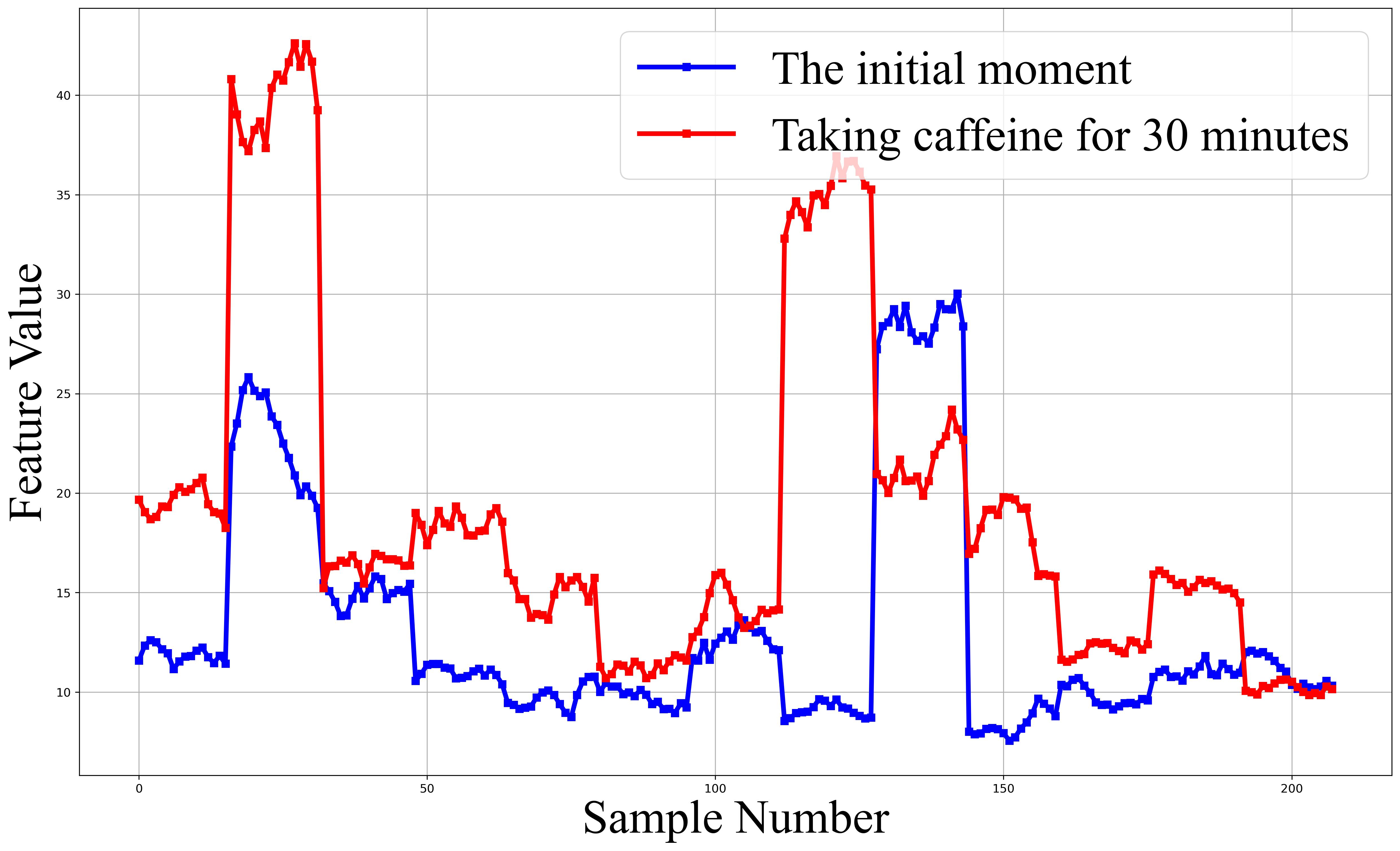

Supplement: Supplementary file 1 [file DataSheet1.ZIP › Suppl.image 15.jpg]

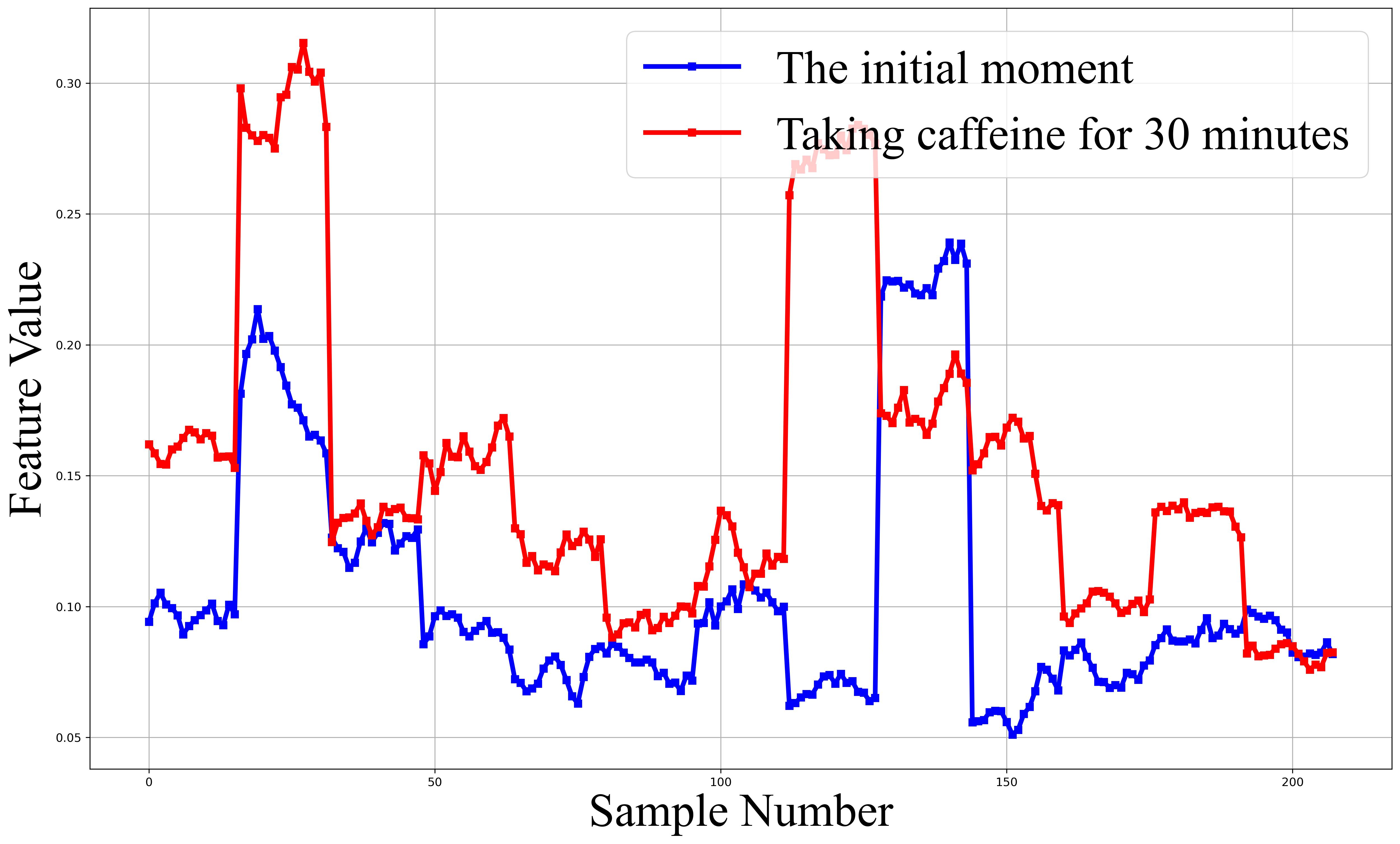

Supplement: Supplementary file 1 [file DataSheet1.ZIP › Suppl.image 16.jpg]

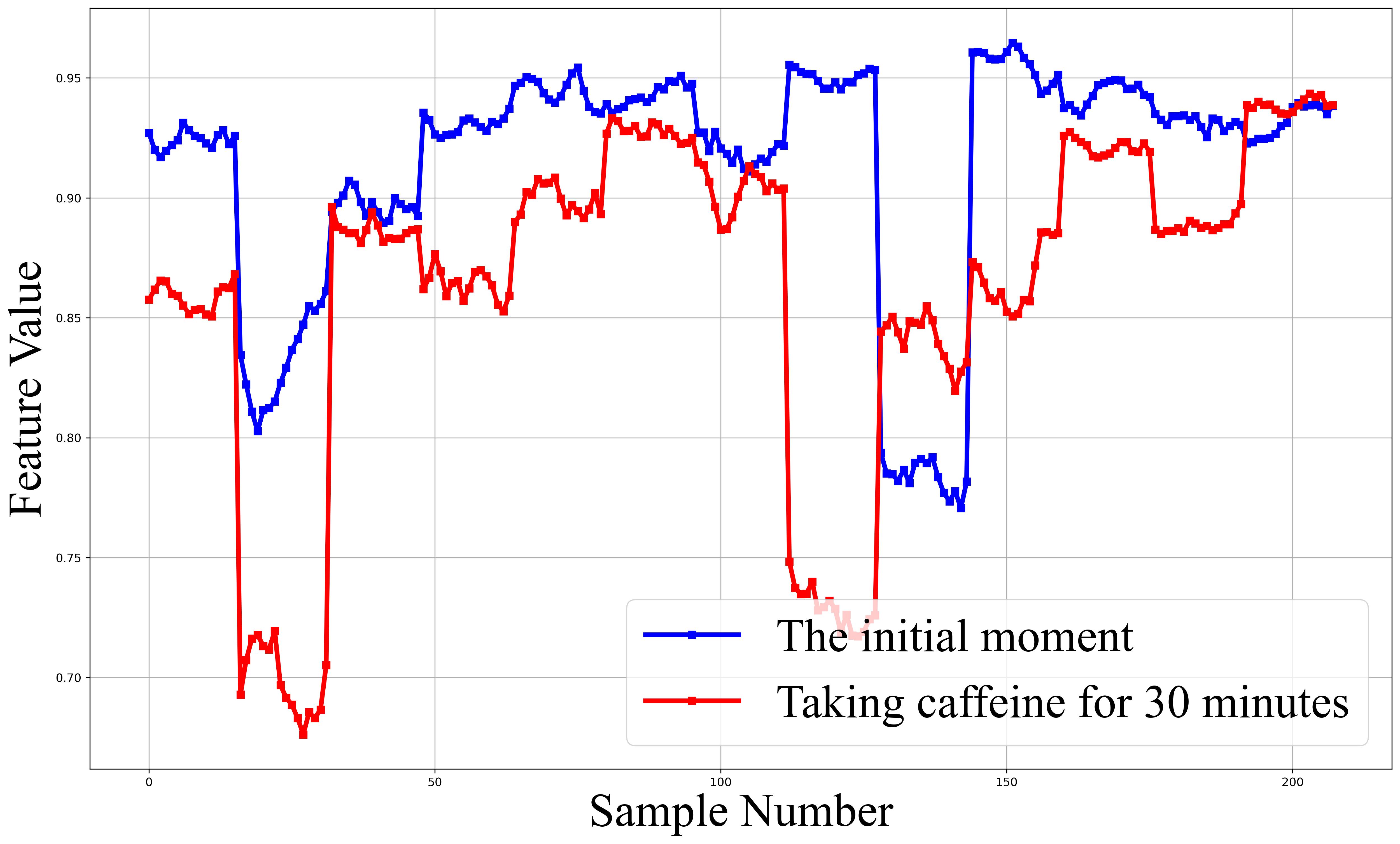

Supplement: Supplementary file 1 [file DataSheet1.ZIP › Suppl.image 17.jpg]

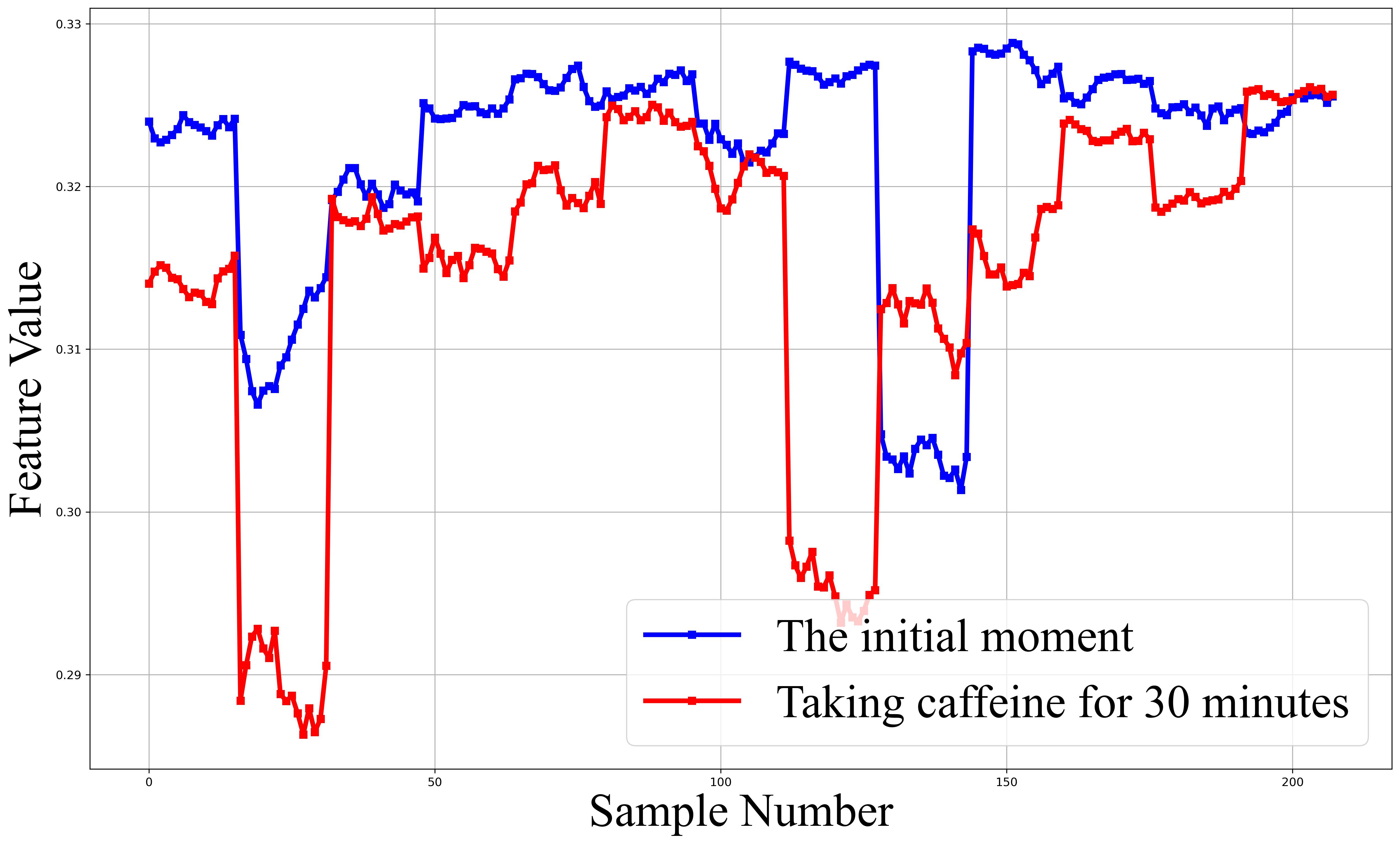

Supplement: Supplementary file 1 [file DataSheet1.ZIP › Suppl.image 18.jpg]

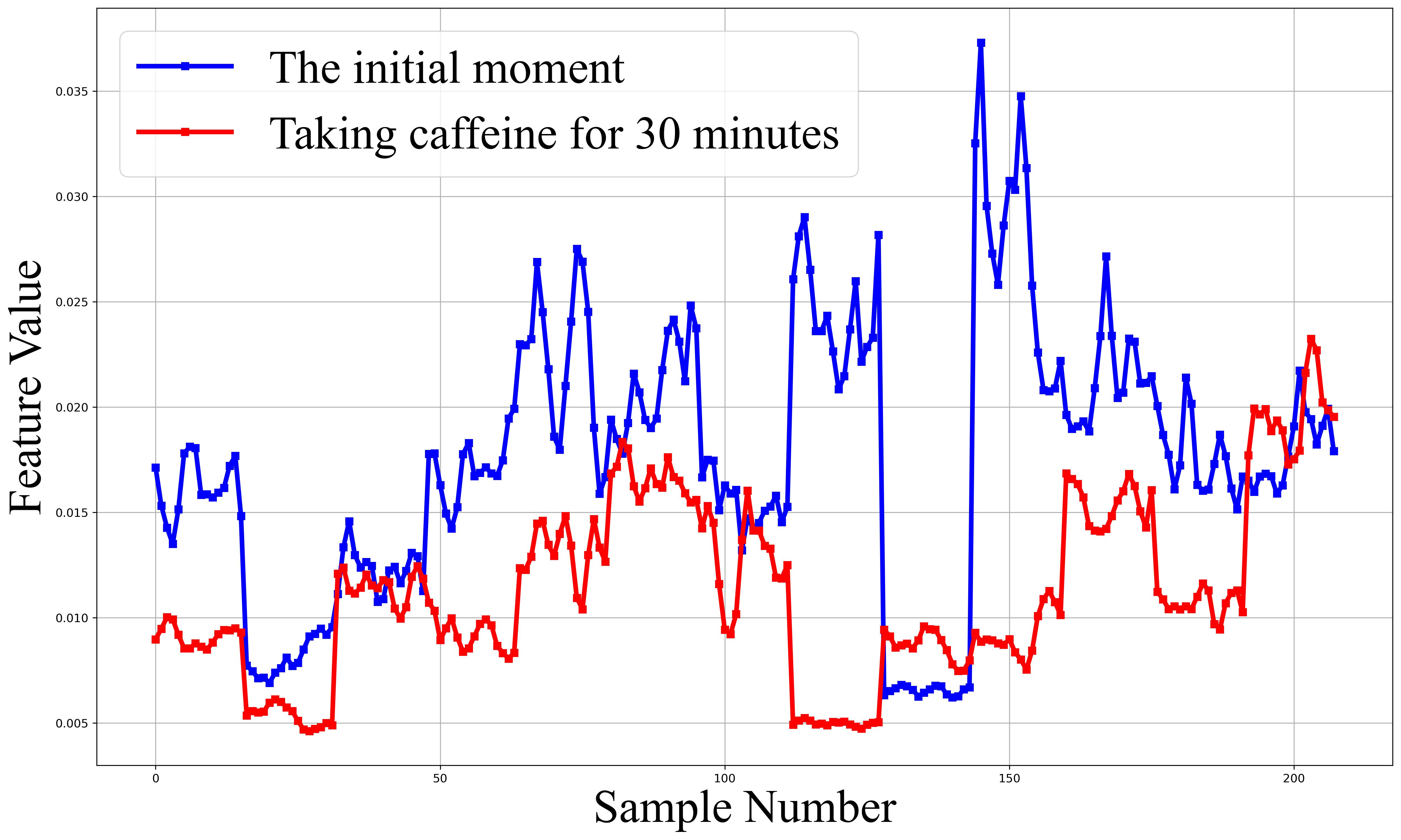

Supplement: Supplementary file 1 [file DataSheet1.ZIP › Suppl.image 19.jpg]

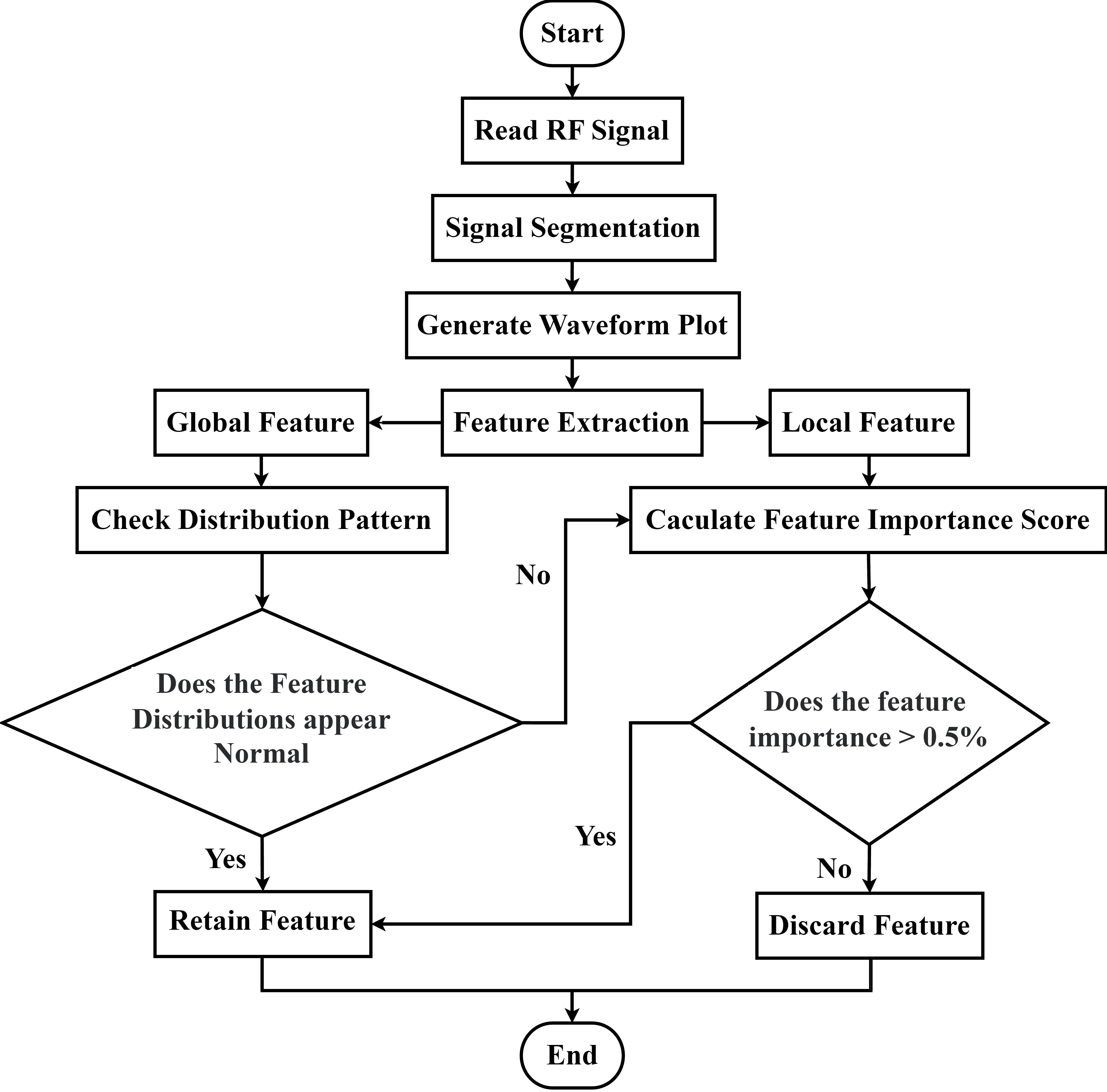

Supplement: Supplementary file 1 [file DataSheet1.ZIP › Suppl.image 2.jpg]

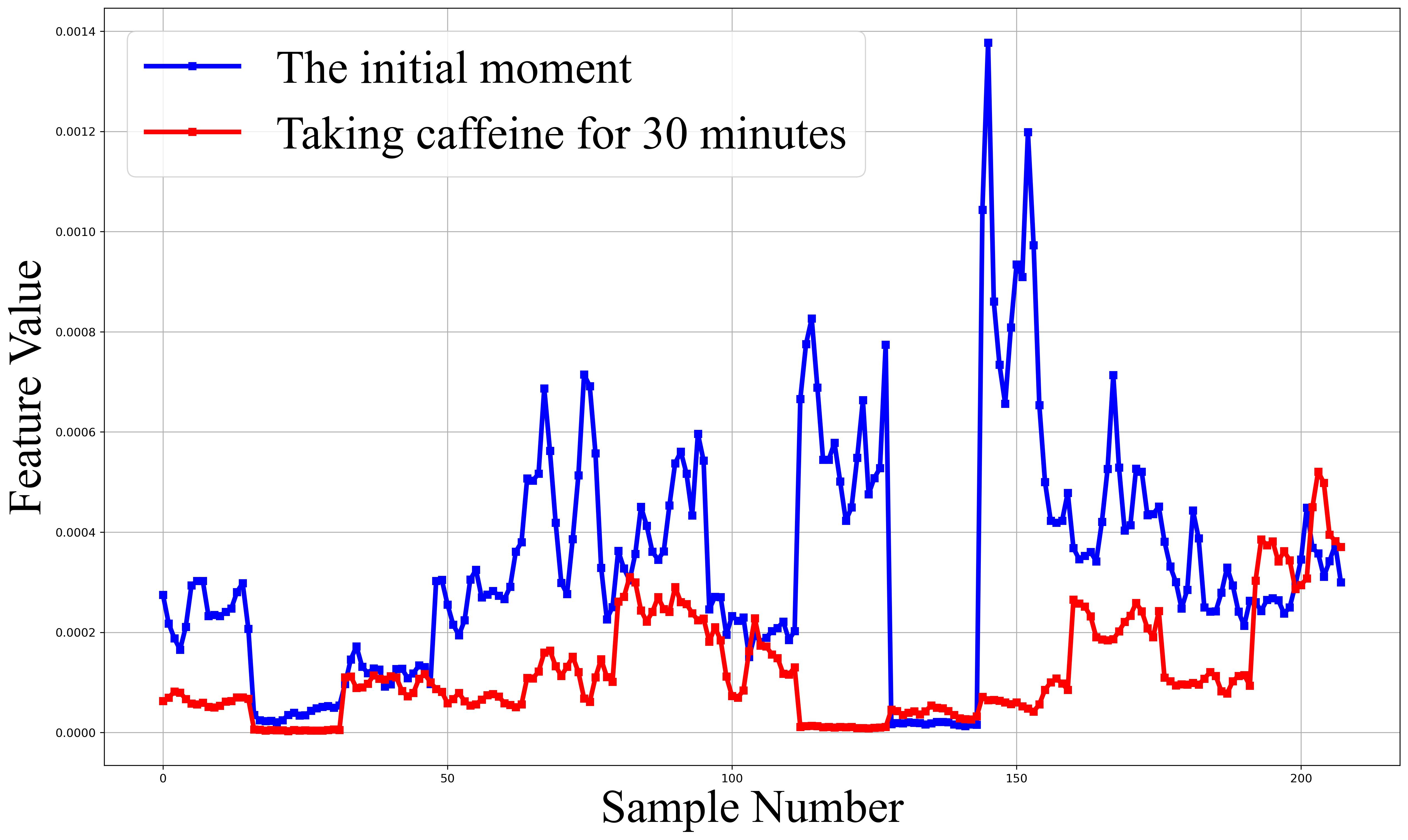

Supplement: Supplementary file 1 [file DataSheet1.ZIP › Suppl.image 20.jpg]

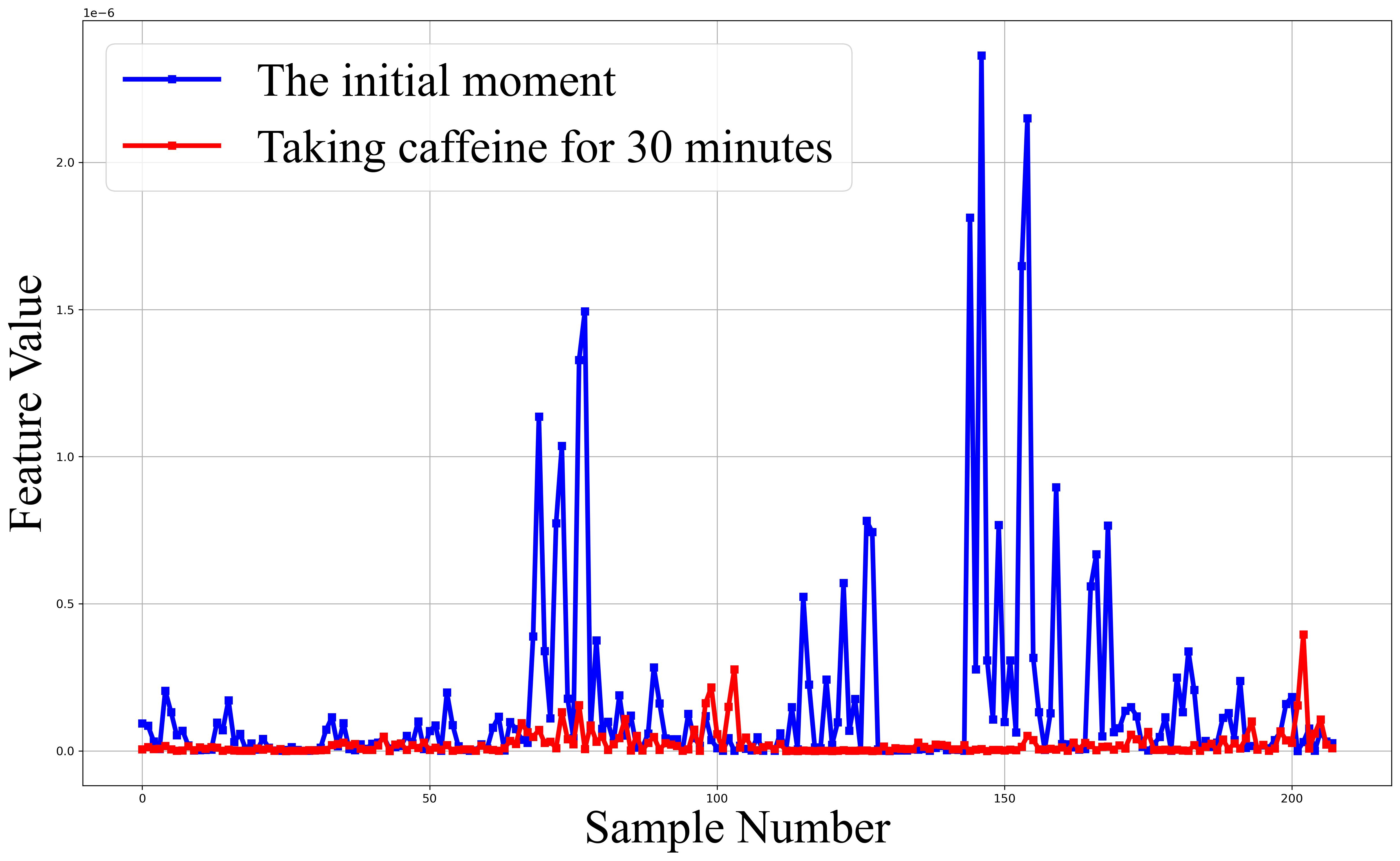

Supplement: Supplementary file 1 [file DataSheet1.ZIP › Suppl.image 21.jpg]

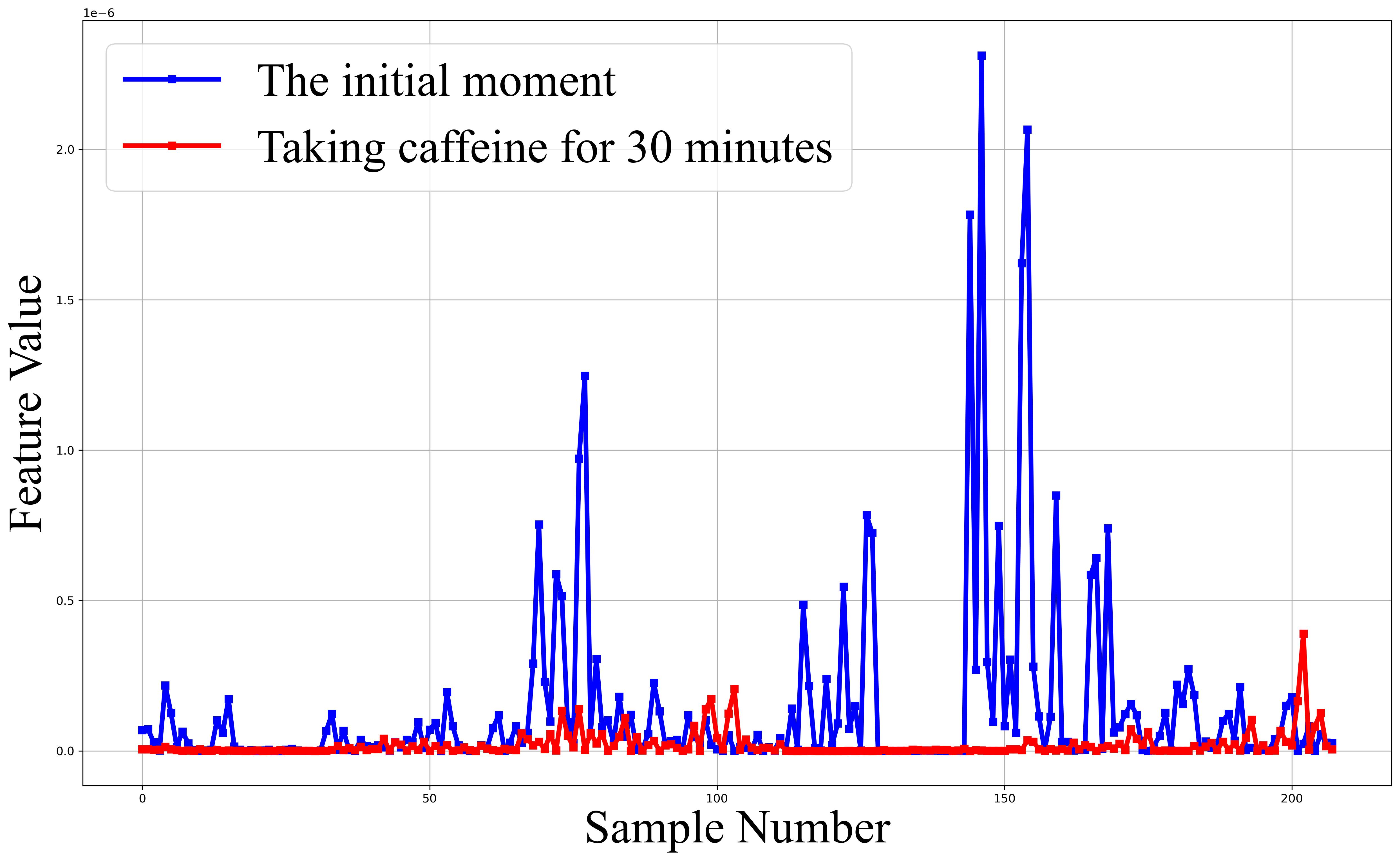

Supplement: Supplementary file 1 [file DataSheet1.ZIP › Suppl.image 22.jpg]

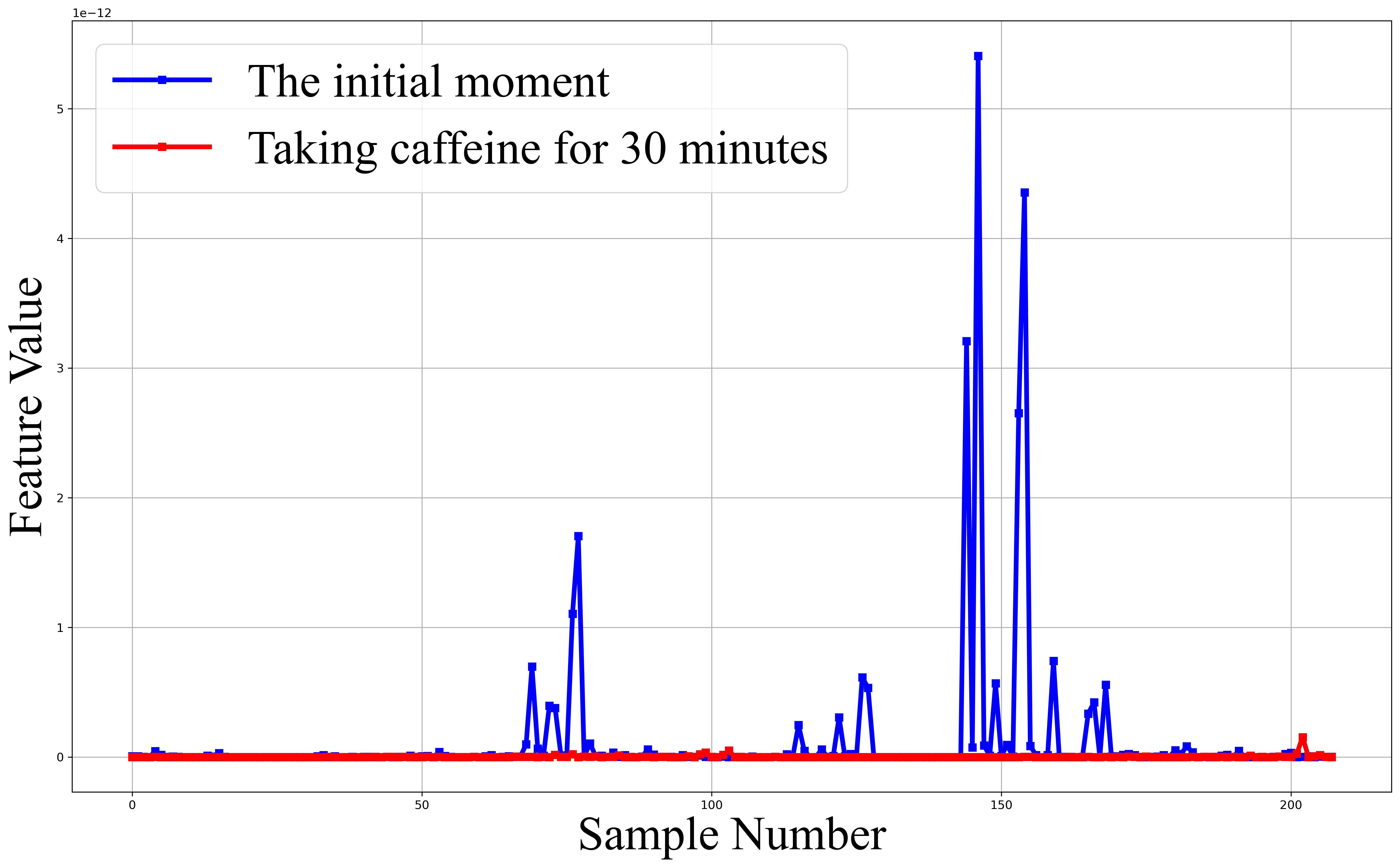

Supplement: Supplementary file 1 [file DataSheet1.ZIP › Suppl.image 23.jpg]

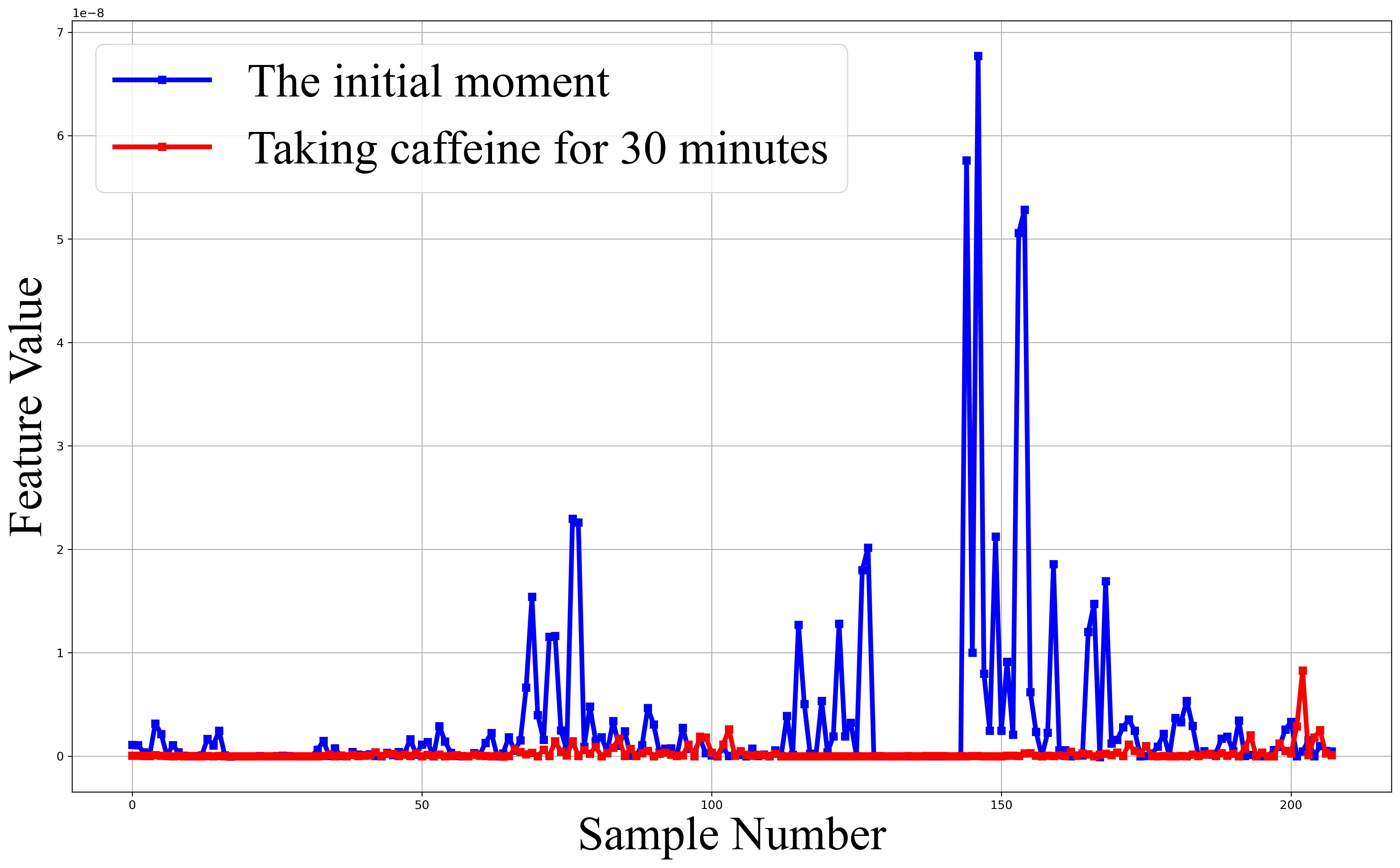

Supplement: Supplementary file 1 [file DataSheet1.ZIP › Suppl.image 24.jpg]

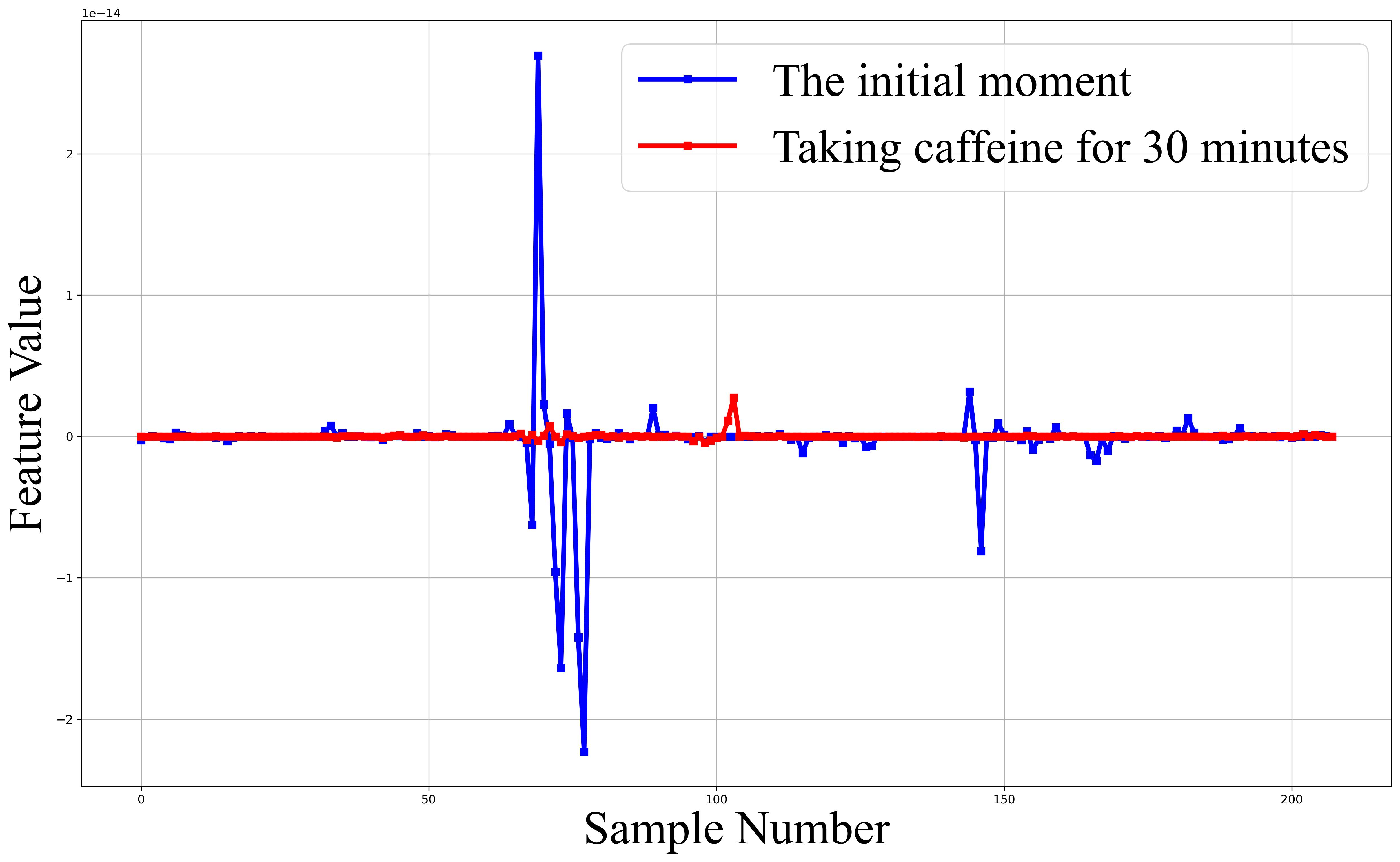

Supplement: Supplementary file 1 [file DataSheet1.ZIP › Suppl.image 25.jpg]

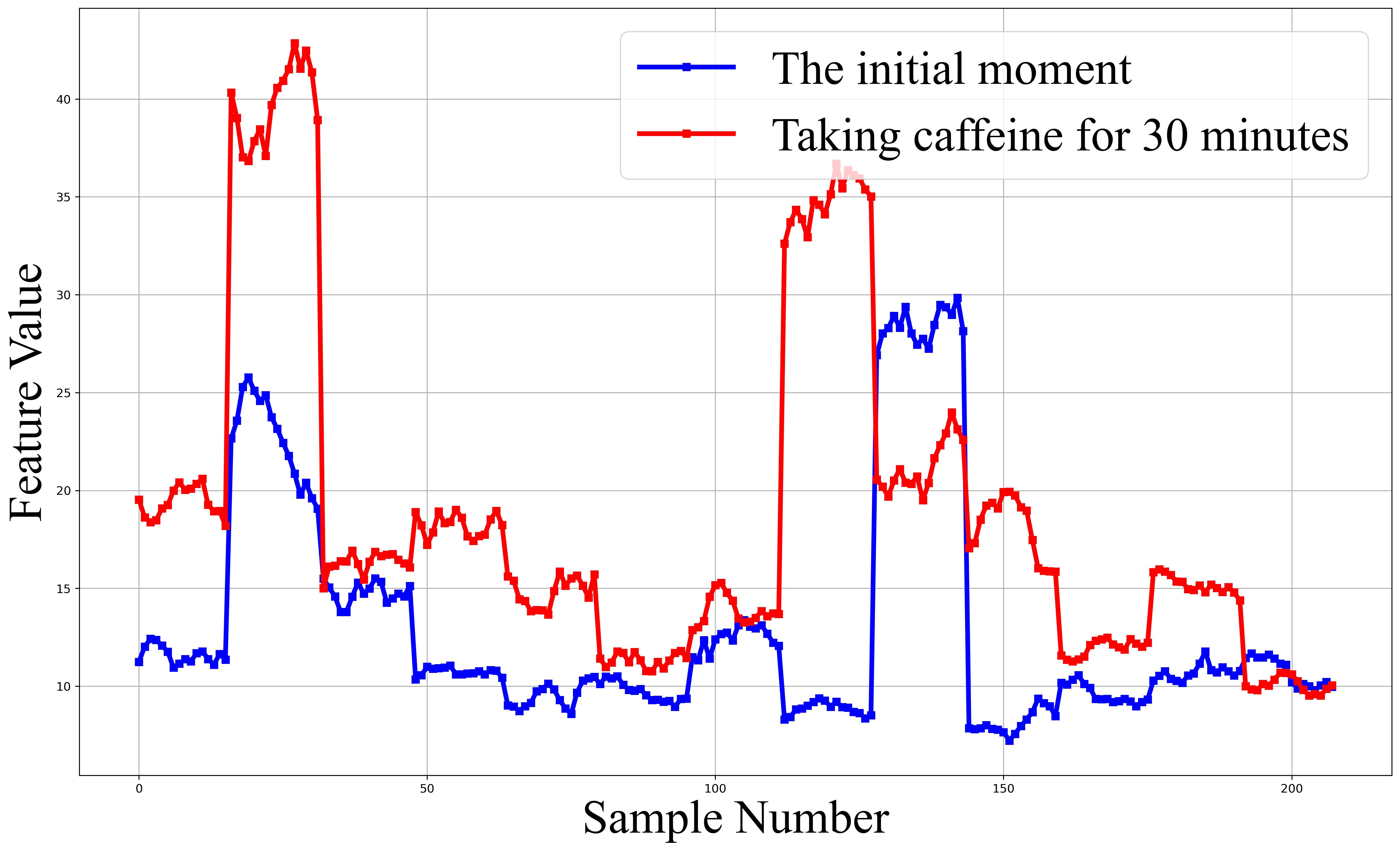

Supplement: Supplementary file 1 [file DataSheet1.ZIP › Suppl.image 3.jpg]

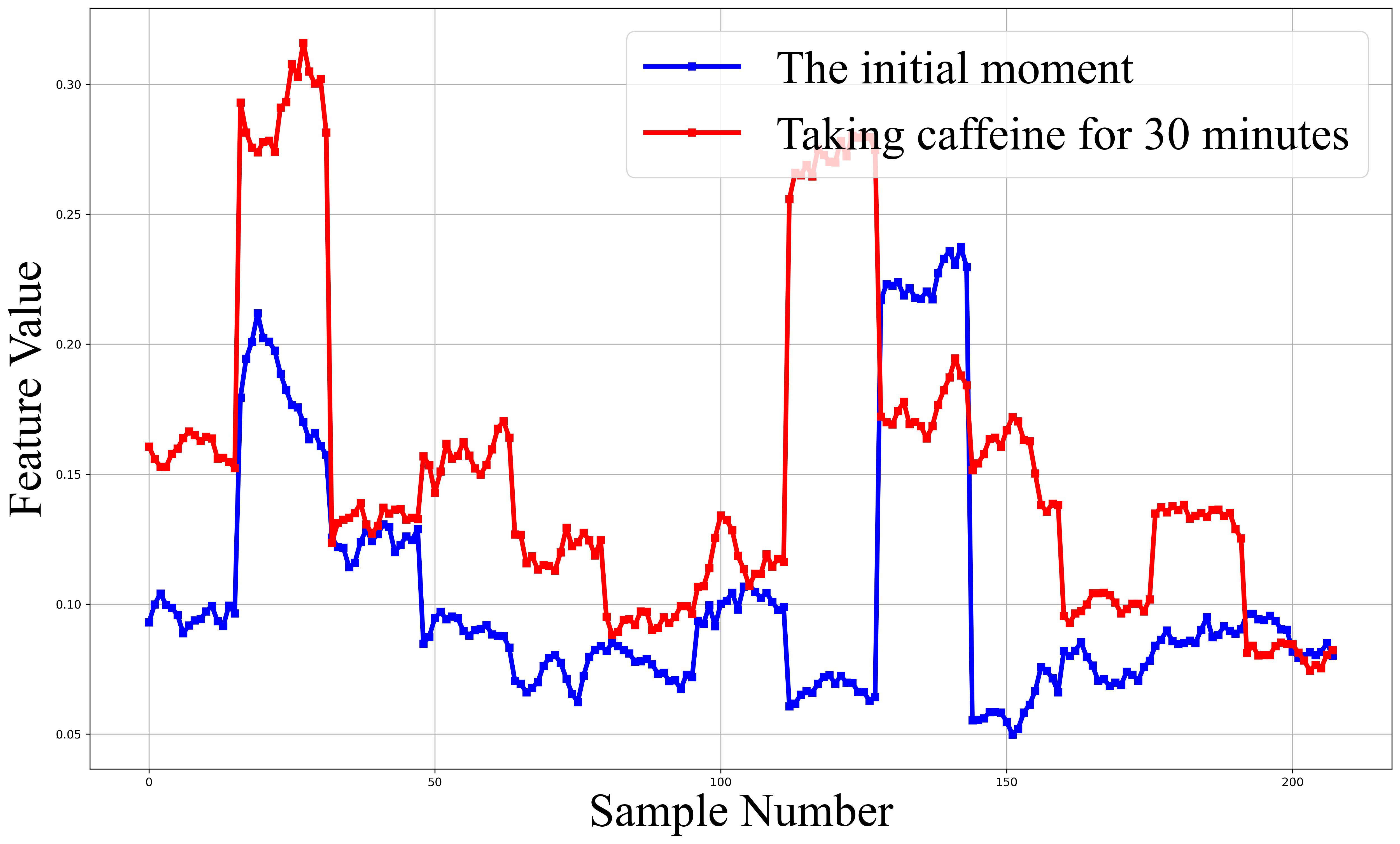

Supplement: Supplementary file 1 [file DataSheet1.ZIP › Suppl.image 4.jpg]

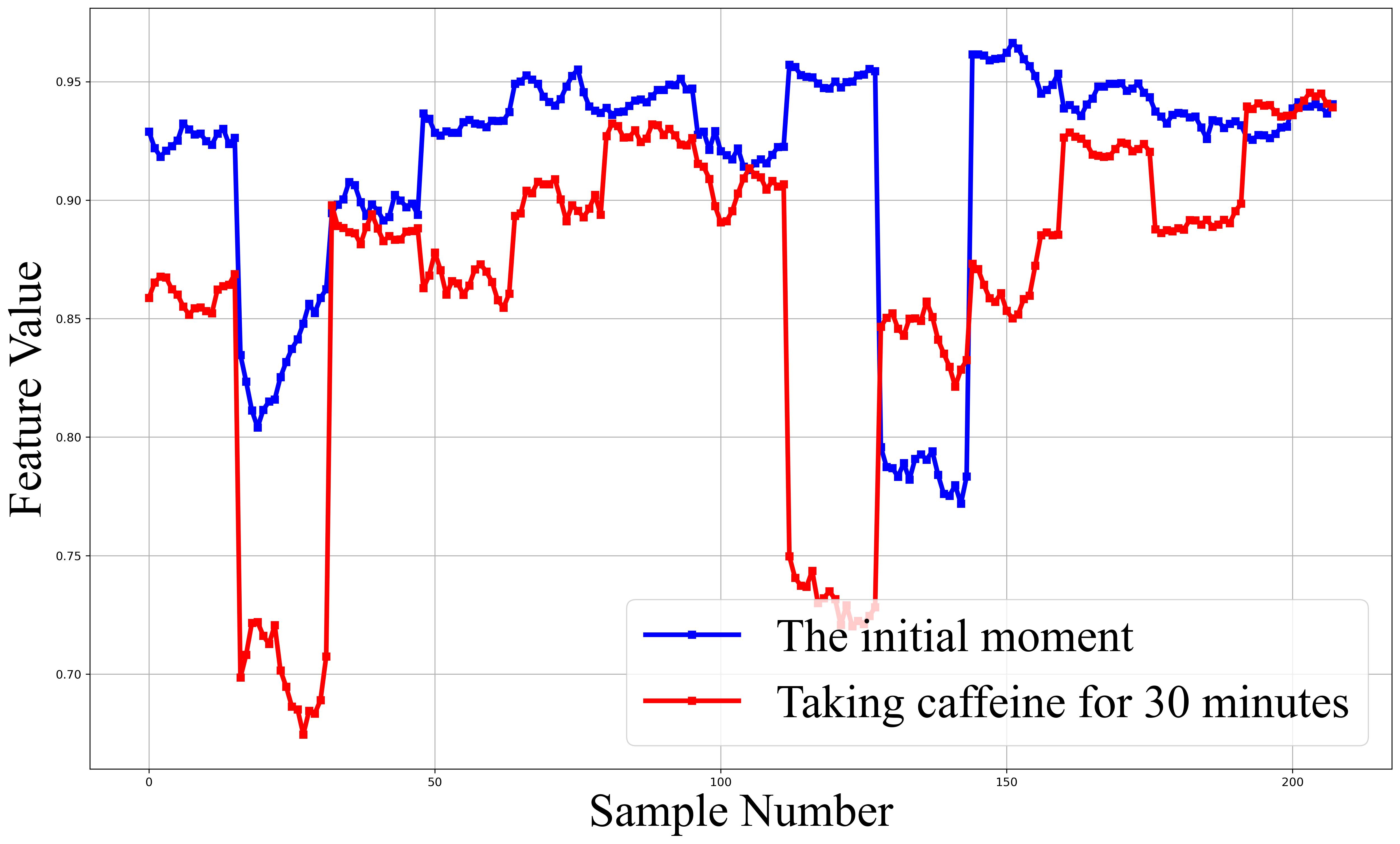

Supplement: Supplementary file 1 [file DataSheet1.ZIP › Suppl.image 5.jpg]

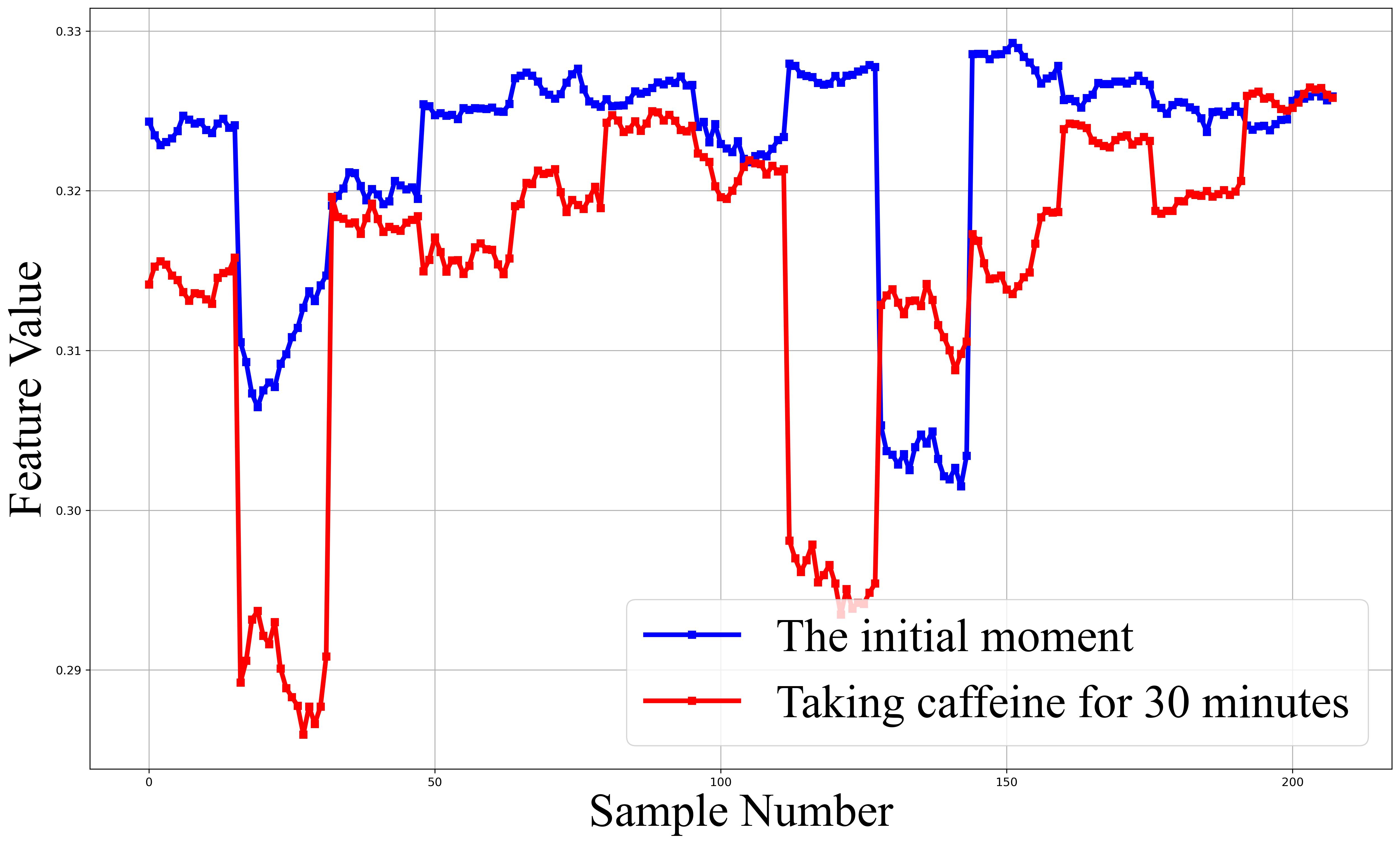

Supplement: Supplementary file 1 [file DataSheet1.ZIP › Suppl.image 6.jpg]

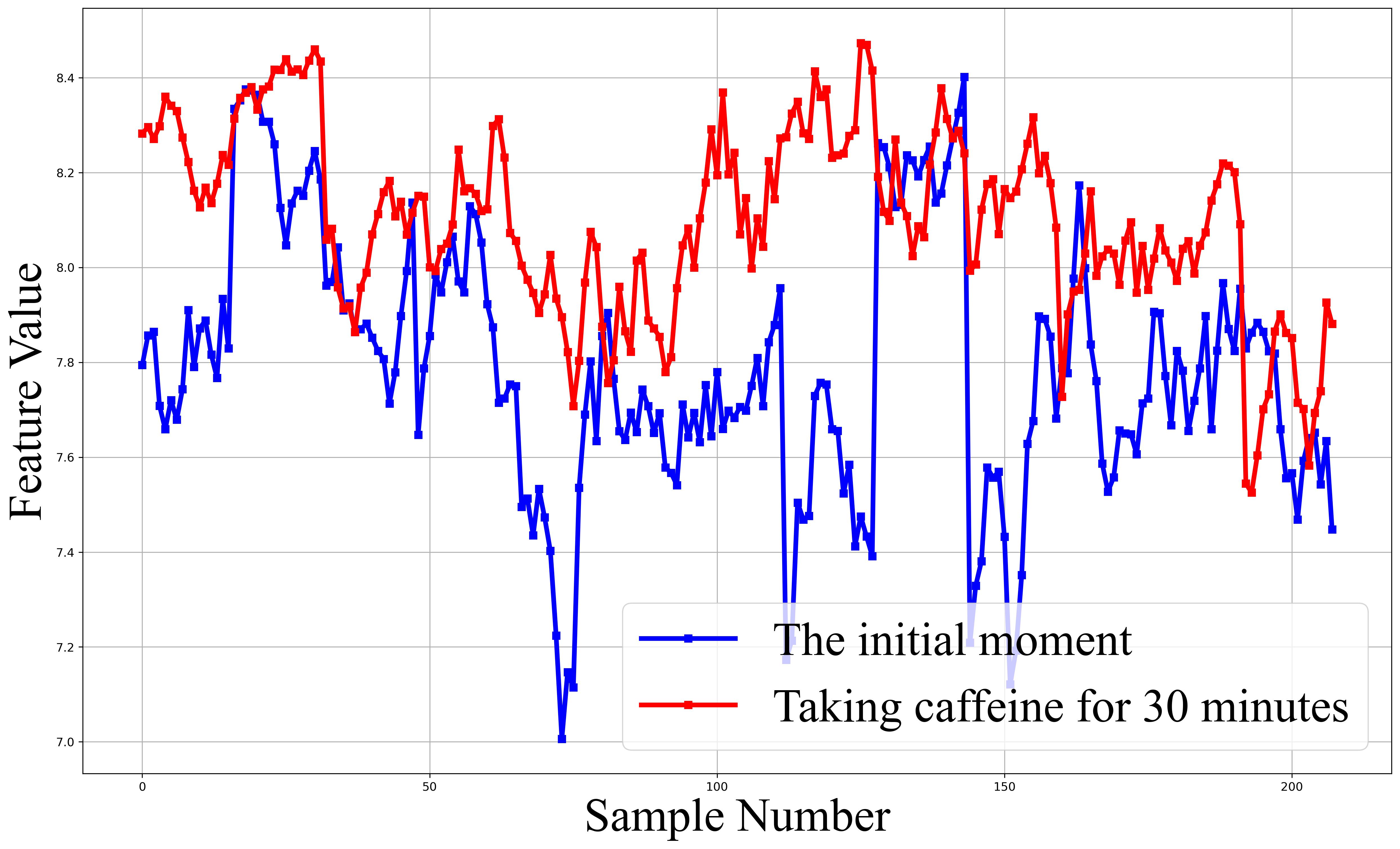

Supplement: Supplementary file 1 [file DataSheet1.ZIP › Suppl.image 7.jpg]

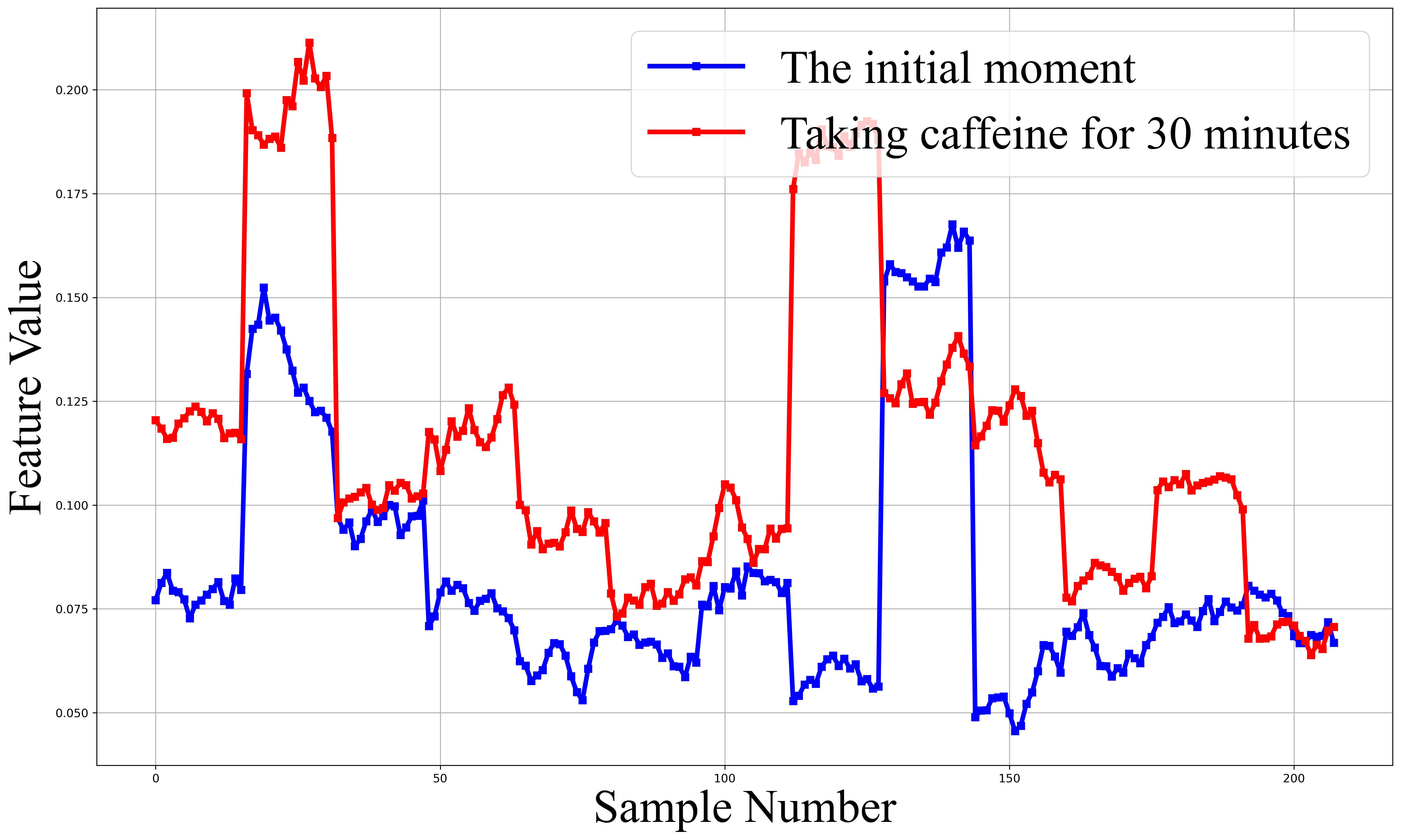

Supplement: Supplementary file 1 [file DataSheet1.ZIP › Suppl.image 8.jpg]

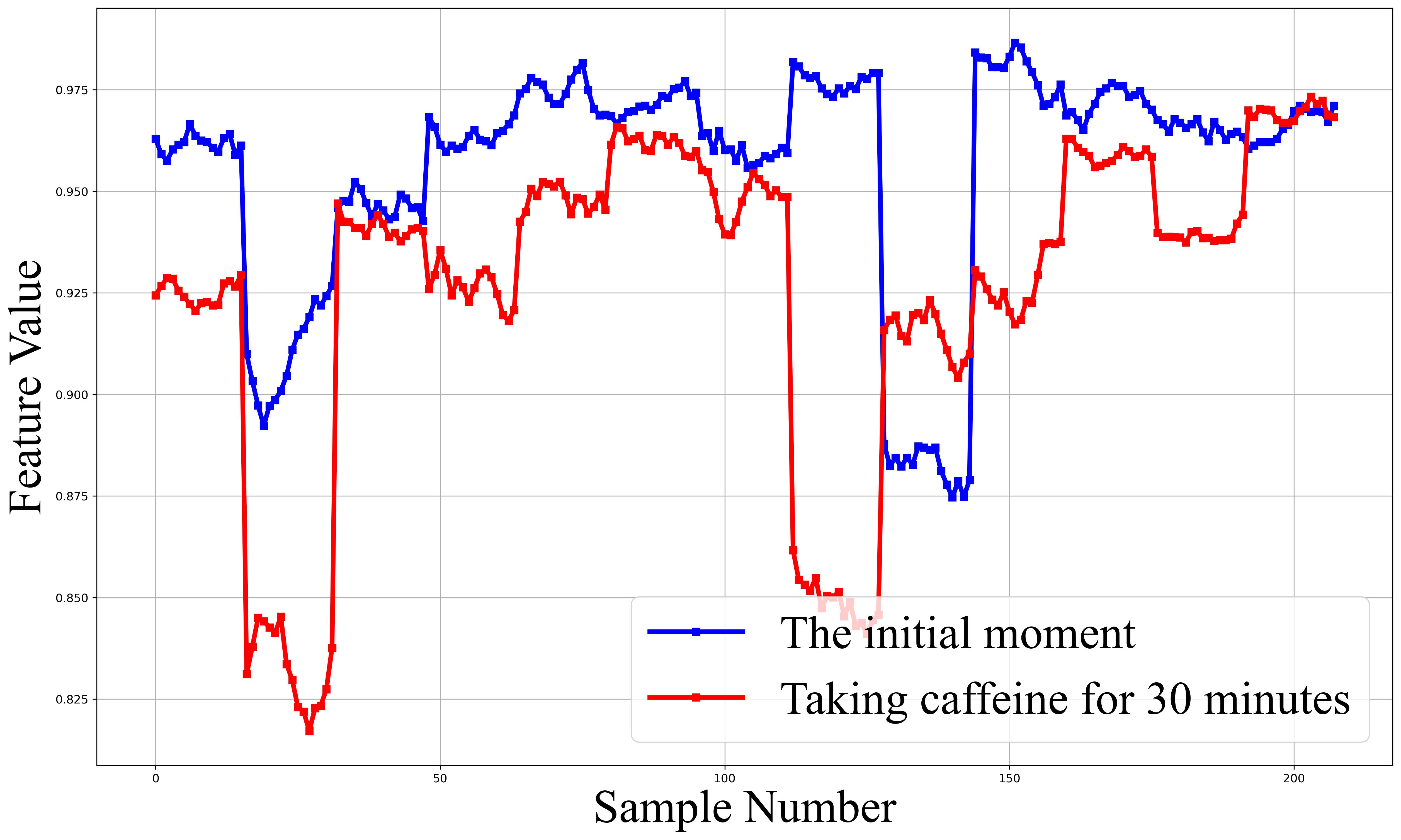

Supplement: Supplementary file 1 [file DataSheet1.ZIP › Suppl.image 9.jpg]
